# Distinct roles of phytochromes A and B in *Aspergillus fumigatus* in environmental sensing and pathogenicity

**DOI:** 10.1128/mbio.02204-25

**Published:** 2025-09-23

**Authors:** Kai Leister, Yan Dong, Alexander Landmark, Yinyan Ma, Birgit Schreckenberger, Zhenzhong Yu, Ling Lu, Reinhard Fischer

**Affiliations:** 1 Department of Microbiology, Institute for Applied Biosciences, Karlsruhe Institute of Technology (KIT)-South Campus 150232, Karlsruhe, Germany; 2 Department of Clinical Laboratory, Nanjing Drum Tower Hospital, College of Life Sciences, Nanjing Normal University 12534, Nanjing, China; 3 Jiangsu Provincial Key Lab for Organic Solid Waste Utilization, Jiangsu Collaborative Innovation Center for Solid Organic Waste Resource Utilization, Educational Ministry Engineering Center of Resource-saving fertilizers, Nanjing Agricultural University 70578, Nanjing, China; University of Melbourne, Melbourne, Victoria, Australia

**Keywords:** phytochrome signaling, *A. nidulans*, *A. fumigatus*, pathogenicity, hybrid histidine kinase, secondary metabolism, neosartoricin B, fumicycline

## Abstract

Phytochromes are red-light receptors that regulate gene expression in response to light and temperature in plants, bacteria, and fungi. In the opportunistic pathogenic fungus *Aspergillus fumigatus*, two putative phytochromes—FphA and FphB—were characterized to assess any roles in virulence. Spectroscopic analysis of *E. coli*-expressed proteins showed that FphA is photoconvertible and functionally similar to its *A. nidulans* ortholog, whereas FphB is photoinactive. Therefore, only FphA could rescue an *A. nidulans fphA*-deletion mutant. Gene deletion in *A. fumigatus* revealed that loss of FphA had no effect on virulence, while deletion of FphB—alone or with FphA—significantly increased virulence in a *Galleria mellonella* infection model. Transcriptomic data linked FphB to the regulation of the neosartoricin B biosynthetic gene cluster and the production of the prenylated nonribosomal peptide hexadehydroastechrome, suggesting a role in regulating secondary metabolism. Localization studies showed FphA at the mitochondria and in nuclei and FphB mainly in the cytoplasm; both proteins form heterodimers in the cytoplasm and in nuclei. These findings suggest that while FphA acts as a light and temperature sensor, FphB modulates virulence and may represent a novel regulatory factor in fungal pathogenicity.

**IMPORTANCE:**

*Aspergillus fumigatus* is a major pathogen in immunocompromised individuals, showing greater virulence than *A. nidulans* despite genetic similarities. A key difference is the presence of two phytochrome-like proteins: FphA, a conserved red-light and temperature sensor, and FphB, a photoinactive hybrid histidine kinase. Notably, FphB appears to suppress virulence, suggesting a regulatory role in signaling pathways that govern pathogenicity and secondary metabolism. We propose FphB functions as a signaling hub linking environmental cues to virulence, with its network offering a promising target for antifungal strategies.

## INTRODUCTION

Phytochromes, well-known red-light photoreceptors in plants, have also been identified in certain bacteria and many filamentous fungi, though they are absent in the Saccharomycotina clade (1–3). Phytochrome sequences were first identified in *Aspergillus fumigatus* and in *Neurospora crassa* and then characterized in *A. nidulans, A. fumigatus,* and *N. crassa* (4–7). Among these, only *A. nidulans* displayed pronounced developmental phenotypes upon phytochrome-gene deletion, including red-light insensitivity and altered balance between asexual and sexual development (5). Genome-wide expression analyses revealed that phytochrome mediates most of the light responses in *A. nidulans* and controls ca. 10% of all genes in the genome (8). In contrast, light responses in *N. crassa* are predominantly mediated by the blue-light receptor WC-1, despite the fact that *N. crassa* contains two phytochromes (9). In *Alternaria alternata,* both phytochrome and a WC-1 ortholog, the blue-light receptor LreA, play roles in light-regulated development and secondary metabolism (10).

 The *A. nidulans* FphA photoreceptor consists of a large protein comprising a photosensory domain (composed of PAS, GAF, and PHY domains) and a C-terminal output domain (comprising a histidine kinase and a response regulator domain) (5). As a difference to plant phytochromes, the histidine kinase domain in the *A. nidulans* phytochrome is still enzymatically active, and a response-regulator domain is fused to the C-terminus (11). The protein most likely binds biliverdin, which probably is produced at the mitochondria (12). FphA was first localized in the cytoplasm, but interaction studies revealed that a fraction of the protein also resides in the nuclei (5, 13). In the cytoplasm of *A. nidulans,* FphA channels the light signal into the HOG pathway by interacting with the phosphotransfer protein YpdA in the cytoplasm (14). This light signaling pathway is conserved in *A. alternata* (10). In the nucleus, the phytochrome interacts with the blue-light photoreceptor complex LreA/LreB, with the velvet transcription factor VeA, and enzymes of the chromatin remodeling machinery (13, 15). Hence, the phytochrome controls gene expression by direct induction of the stress pathway in the cytoplasm with the MAP kinase SakA (HogA) and the transcription factor AtfA as central components, through interaction with transcriptional regulators in the nucleus and through chromatin remodeling.

 Besides light signaling, there is also evidence for the temperature-sensing function of phytochromes *in planta* and in *A. nidulans* and *A. alternata* (16–18). Whereas in plants the temperature modulates the light response of phytochrome, the two fungal phytochromes sense ambient temperatures in the dark.

 The opportunistic pathogen *A. fumigatus* harbors two phytochromes, one of which was analyzed by gene deletion. It was named FphA because it was more similar to *A. nidulans* FphA than the second *A. fumigatus* paralog. Despite the sequence similarity between AfFphA and AnFphA, deletion of *fphA* in *A. fumigatus* had minor light-dependent phenotypes (6, 19). Only the germination process appeared to be affected. Although germination was inhibited by light, deletion of *fphA* caused slower germination under dark conditions. This phenotype is shared in *A. nidulans* and suggests functions of the phytochrome in the dark (20). The second phytochrome of *A. fumigatus* has not been studied in detail yet. Both phytochromes were classified as class VIII hybrid histidine kinases, and *A. fumigatus* contains thirteen of such enzymes (21).

In *A. nidulans*, the phytochrome functions as both a red-light and temperature sensor. Given that temperature sensing occurs in the dark, it is plausible that this was the ancestral role of phytochromes, with light sensing evolving later through the acquisition of a chromophore. The presence of two phytochromes in *A. fumigatus* led us to hypothesize that one may act as a light sensor, while the other mediates temperature sensing. Another motivation for this study was molecular evidence from *A. nidulans* showing that the phytochrome also performs light-independent functions beyond temperature sensing (8). Since fungal infection occurs in darkness, any role of phytochromes in pathogenicity would likely depend on their dark functions. Our findings support this: one phytochrome (FphA) is a functional light and temperature sensor, while the second (FphB) likely acts as a hybrid histidine kinase that regulates secondary metabolite gene clusters and attenuates virulence.

## RESULTS

### Spectroscopic characterization of two phytochromes from *A. fumigatus*

**Fig 1:**
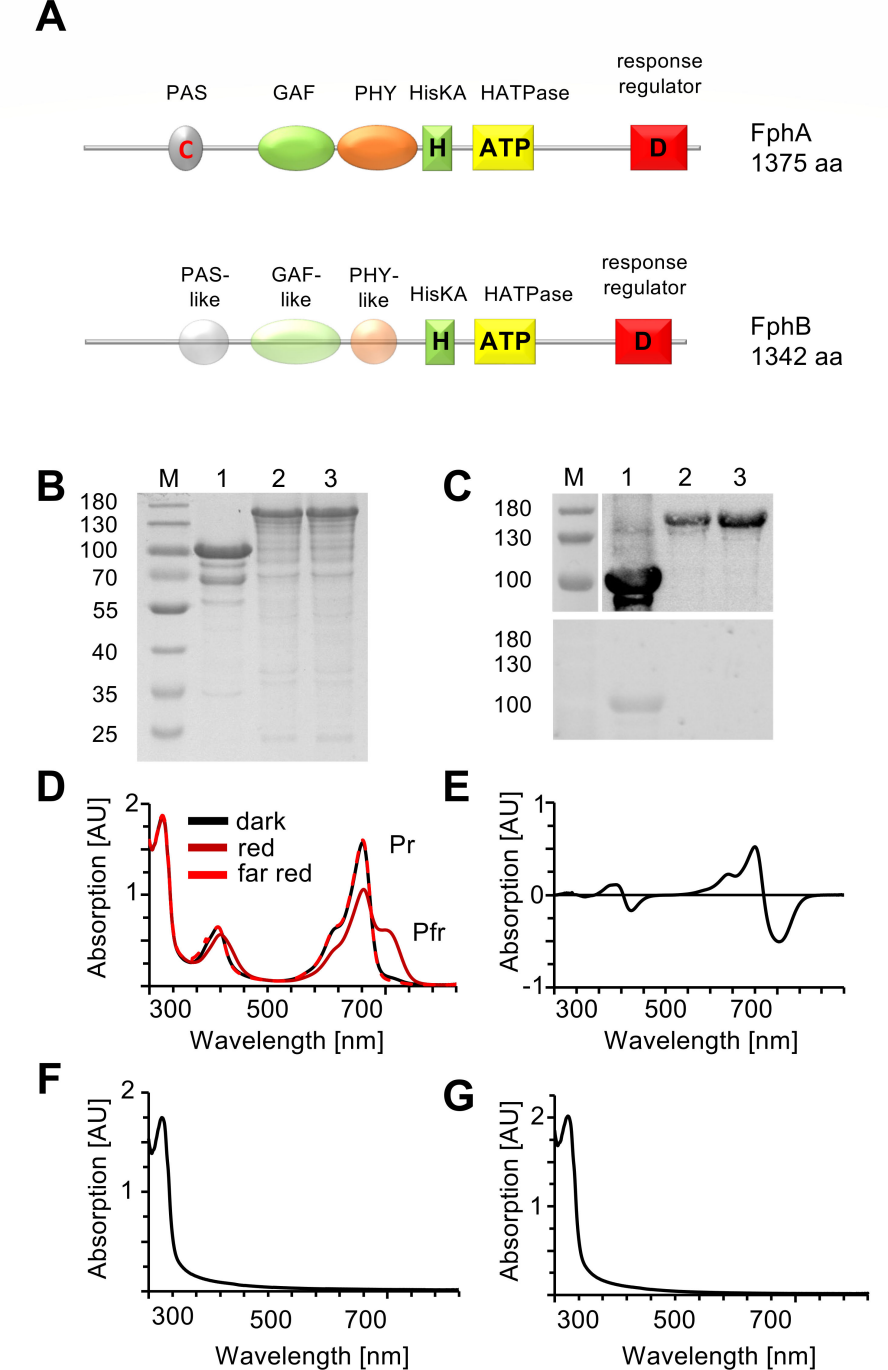
Spectroscopic characterization of two putative phytochromes from *A. fumigatus*. (**A**) Schematic domain arrangement of FphA and FphB. PAS: PER-ARNT-SIM domain. GAF: cGMP-adenylyl cyclase-FhlA domain. PHY: phytochrome domain. HisKA: histidine kinase A. (**B**) SDS-PAGE of purified phytochromes of *A. fumigatus*, stained with Coomassie Brilliant Blue. Lanes: M, PageRuler prestained protein ladder, 10–180 kDa; 1, AfFphA-NPGP (photosensory domain); 2, AfFphB; 3, AfFphB^M203C^. (**C**) (Top) Western blot of the purified phytochromes. The strep-tagged proteins were detected using StrepMAB-Classic HRP-conjugated monoclonal antibodies (IBA Lifesciences, Göttingen, Germany). (Bottom) Zinc-induced red fluorescence of the purified phytochromes. Proteins were separated in a polyacrylamide gel containing zinc acetate. Linear tetrapyrroles form a complex with zinc ions that can be excited by UV light. Lanes: M, PageRuler prestained protein ladder, 10–180 kDa; 1, AfFphA-NPGP (photosensory domain); 2, AfFphB; 3, AfFphB^M203C^. (**D**) UV/vis absorption spectrum of AfFphA-NPGP (photosensory domain) in the dark and after illumination in the Pr or the Pfr form. (**E**) Difference spectrum of AfFphA-NPGP (Pr-Pfr). (**F**) UV/vis absorption spectrum of AfFphB in the dark. (**G**) UV/vis absorption spectrum of AfFphB^M203C^.

Bioinformatic analyses of AfFphA and AfFphB showed that both contain a histidine kinase/ATPase and a response regulator domain in their C-terminal output module (Fig. 1A; Fig. S1). However, AfFphB is lacking the critical cysteine required for chromophore binding in the PAS domain and motifs in the GAF and PHY domain that are involved in the transduction of the light signal to the output module of the protein (Fig. 1A; Fig. S2), suggesting that only FphA acts as a photoreceptor. Instead of a cysteine at the critical position, a methionine is present, which also provides a thiol group like cysteine. In comparison, the two phytochromes of *N. crassa* and the three phytochromes of *B. cinerea* all contain the critical cysteine for chromophore binding. To test the hypothesis that only *A. fumigatus* FphA is photoactive, we co-expressed AfFphA and AfFphB with the bacterial heme oxygenase BphO from *Pseudomonas aeruginosa* in *Escherichia coli*, purified the two strep-tagged proteins, and studied their spectroscopic properties. FphB could be expressed as full-length protein, whereas the FphA full-length protein was insoluble. Therefore, only the photosensory module of FphA was produced (Fig. 1B and C). AfFphA showed an absorption spectrum of a typical phytochrome with absorption maxima of the Q-band at 702 nm in its Pr state and 754 nm in its Pfr state and of the Soret band around 400 nm (Fig. 1D and E). FphB did not absorb red or far-red light (Fig. 1F). Next, we tested if replacement of the methionine to cysteine into the conserved region (M203) of the photosensory module would restore the photoconvertibility of the protein. However, the FphB^M203C^ protein was still inactive (Fig. 1G). To test for chromophore binding, the proteins were separated by SDS-PAGE containing zinc acetate (Fig. 1C lower panel). Linear tetrapyrroles form fluorescent complexes with zinc ions that can be excited by UV light. FphA showed a signal in UV light, whereas FphB and the FphB^M203C^ mutant did not. If FphA behaves like a red-light photoreceptor *in vitro*, we anticipated that AfFphA could rescue an *AnfphA*-deletion strain in *A. nidulans*. FphB, in contrast, could be involved in temperature sensing.

### *A. fumigatus* phytochrome A can act as a photo sensor and as temperature sensor in *A. nidulans*

**Fig 2:**
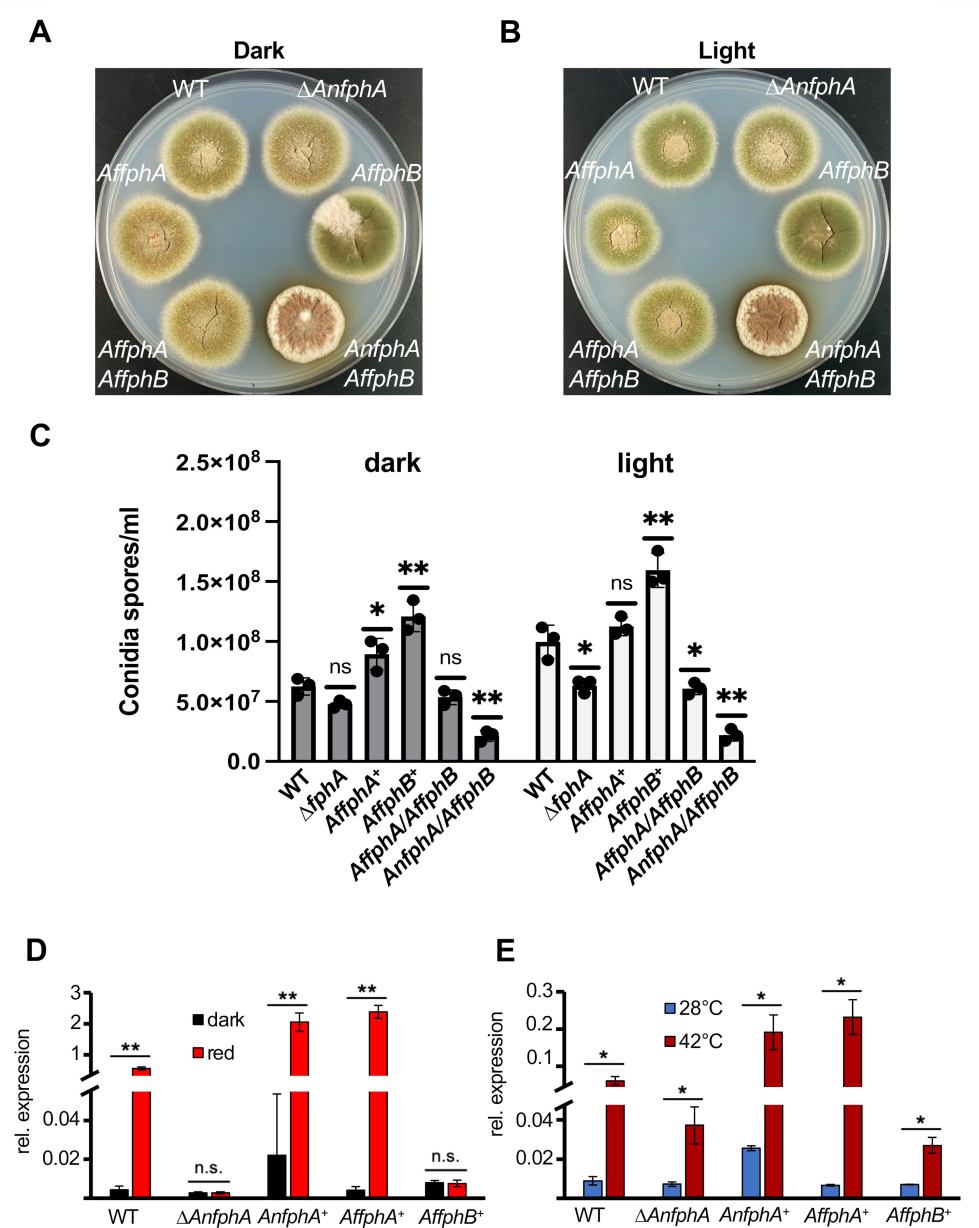
*A. fumigatus* phytochrome A complements the photo- and the temperature-sensing functions of AnFphA in *A. nidulans*. (**A**) Colony phenotypes in the dark of *A. nidulans* WT, ∆*fphA,* ∆*fphA* complemented with *A. fumigatus fphA* and/or *fphB,* and *A. nidulans* expressing *AnfphA and AffphB*. (**B**) Colony phenotypes in light of *A. nidulans* WT, ∆*fphA,* ∆*fphA* complemented with *A. fumigatus fphA* and/or *fphB,* and *A. nidulans* expressing *AnfphA* and *AffphB*. (**C**) Quantification of conidia in the strains indicated. Spores (1 × 10^5^) were inoculated on solid YAG media and incubated for 3 days either in full darkness (dark) or white light (light). Spores were harvested and counted in a Neubauer chamber. Statistical significance of mutant strains refers to WT in the respective condition. Error bars represent the standard deviation of three biological replicates and two technical replicates. For statistical analysis, a two-tailed Student’s *t*-test was performed: ns, *P* > 0.05; *P ≤ 0.05; **P ≤ 0.01. Dots represent each individual biological replicate. (**D**) Induction of *ccgB* after treatment with red light for 15 min of the strains expressing either *fphA* or *fphB* from *A. fumigatus*. Gene expression has been normalized to *h2b*. (**E**) Induction of *ccgB* after temperature shift of the strains expressing either *fphA* or *fphB* from *A. fumigatus*. Gene expression has been normalized to *h2b*. Three biological replicates and technical duplicates were used for RT-qPCR experiments. *t*-test (two-tailed, heteroscedastic): ***P ≤ 0.001; **P ≤ 0.01; *P ≤ 0.05; n.s. (not significant) *P* > 0.05.

We analyzed the functions of AfFphA and AfFphB as light and/or temperature sensors in *A. nidulans*. First, we tried to complement an *AnfphA-*deletion strain and analyzed developmental phenotypes. The genes of *AffphA* and *AffphB* were fused to the promoter region of *AnfphA* and integrated ectopically in the genome of the *A. nidulans fphA*-deletion strain. As a positive control, *A. nidulans* was complemented with the *A. nidulans fphA* gene. *A. nidulans* WT grows in the dark to the same colony size as in light, but the balance between asexual and sexual development is different in dark and light (Fig. 2A and B). In the dark, less conidia are produced, and the sexual cycle is initiated in many places. Therefore, the colony appears greenish with yellow spots (accumulations of Hülle cells, the initials of the sexual cycle). In comparison, in light, the sexual cycle is largely repressed, giving rise to more green-appearing colonies. The *fphA-*deletion strain is “blind,” and hence yellow sexual structures are also produced in light. The colonies grown in light or dark look very similar. The *A. nidulans* strain expressing *A. fumigatus* FphA or FphA and FphB rescued the phenotype of the *fphA* deletion, and colonies appeared more yellow in the dark than in light. When *fphB* was expressed in the *fphA-*deletion strain, more conidia were produced in the dark and in light, suggesting stimulation of the asexual pathway or inhibition of the sexual pathway also in the dark (Fig. 2C). In the presence of *A. nidulans* FphA and additional expression of *A. fumigatus* FphB, a strong developmental phenotype was observed. Almost no asexual conidia were produced, and the sexual cycle appeared to be inhibited. These results suggest that AfFphB interferes with the functions of AnFphA.

Next, we aimed at studying the effects of *A. fumigatus* FphA and FphB expression in *A. nidulans* ∆*fphA* at the molecular level. The complemented strains with the *fphA* genes from both *A. nidulans* and *A. fumigatus* showed light-dependent upregulation of *ccgB*, whereas the complementation with *AffphB* showed no upregulation of the gene (Fig. 2D). Next, we tested whether AfFphB or also AfFphA could substitute for the AnFphA temperature-sensing activity and monitored *ccgB* expression in response to temperature shifts (28°C and 42°C). The magnitude of *ccgB* induction due to temperature shifts was significantly lower than due to light, resulting in higher variances. It has to be considered that in addition to phytochrome, other proteins like TcsB or the blue-light receptor LreA are involved in temperature-dependent gene induction (16, 22). Therefore, *ccgB* induction in the *A. nidulans fphA*-deletion strain was only reduced compared to wild type. The AfFphB-expressing strain in the ∆*fphA* background showed a reduction of *ccgB* expression upon temperature increase similar to the *A. nidulans fphA*-deletion strain. AfFphA restored the temperature-dependent induction of *ccgB* (Fig. 2E).

Both the results of the *in vitro* and the *in vivo* experiments show that AfFphA has the potential to fulfill both the photoreceptor and the temperature sensor function of *A. nidulans* FphA. AfFphB appears to have no function in light nor in temperature sensing.

### Phytochrome A is involved in cell wall homeostasis, and overexpression reduces the growth rate at lower temperatures in *A. fumigatus*

**Fig 3:**
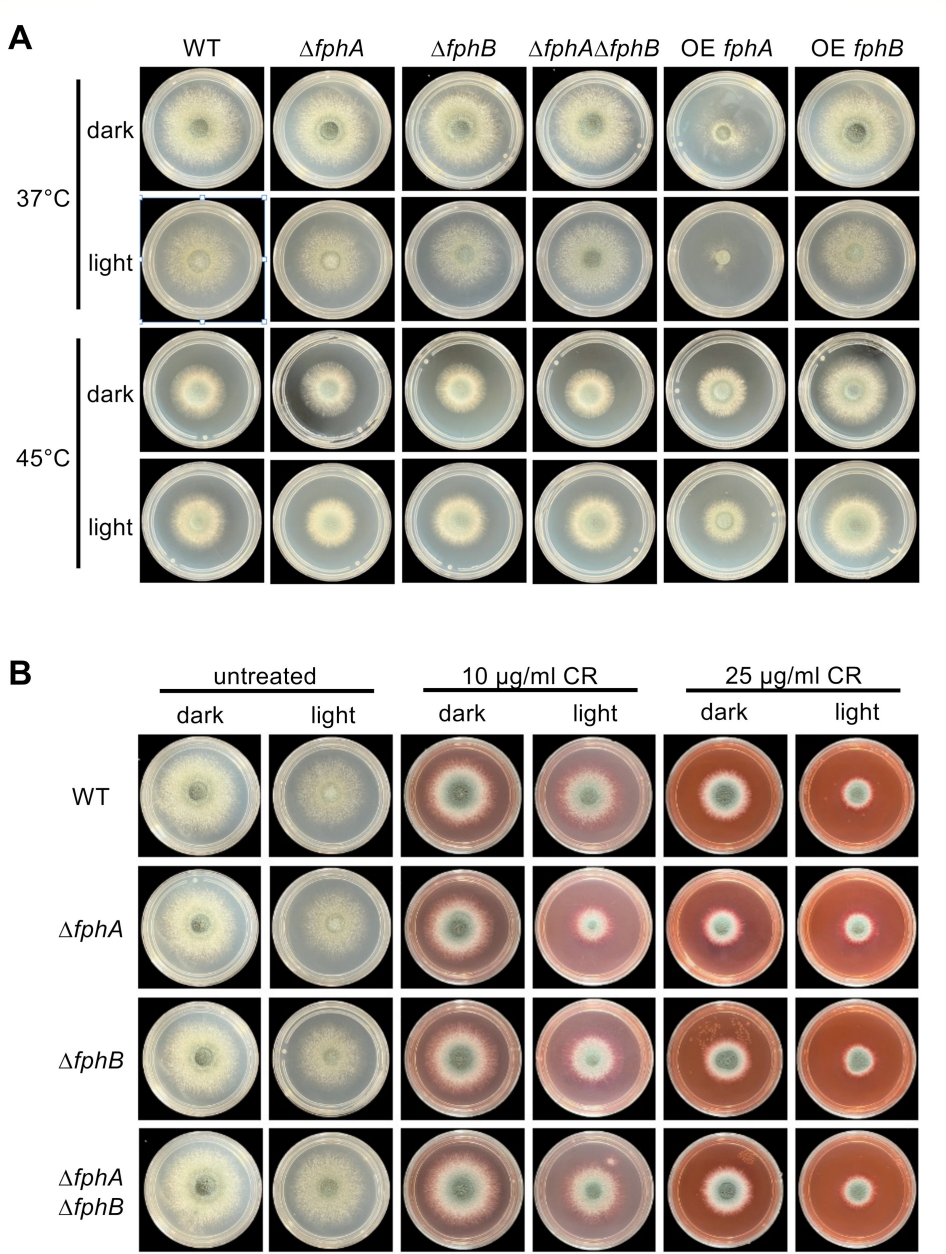
*A. fumigatus* FphA is involved in cell wall homeostasis, and overexpression causes a strong developmental phenotype. (**A**) Colony phenotypes of *A. fumigatus* WT, ∆*fphA,* ∆*fphB,* ∆*fphA*∆*fphB,* OE *fphA,* and OE *fphB* at 37°C and 45°C. OE *fphA* causes a strong developmental phenotype at 37°C that can be rescued at 45°C. Strains were grown on minimal medium for 48 hours at the indicated temperatures in the dark and in light. (**B**) Colony phenotypes of *A. fumigatus* WT, ∆*fphA,* ∆*fphB,* and ∆*fphA*∆*fphB* under the influence of Congo red (10 µg/mL and 25 µg/mL). Strains were grown for 48 hours at 37°C.

After the initial characterization of AfFphA and AfFphB, we analyzed their role in *A. fumigatus* and created corresponding deletion and double-deletion strains (Fig. S3). Neither deletion of *fphA* or *fphB* nor the deletion of both affected vegetative growth, whereas overexpression of *fphA* reduced radial growth of *A. fumigatus* on minimal medium at 37°C in the dark and in light. Interestingly, this inhibitory effect on radial growth was rescued at 45°C (Fig. 3A). Overexpression of *fphB* had no effect. Because FphA uses the HOG pathway for signal transduction and because deletion of *hogA* in *A. alternata* leads to severe growth defects, one hypothesis is that the effect of FphA on growth depends on misregulation of the HOG pathway (10).

Next, we tested if deletion of *fphA, fphB,* or deletion of both genes influences cell wall homeostasis during growth on minimal medium containing Congo red. The *fphA*-deletion strain grew slower on agar plates with Congo red in light (10 µg/mL Congo red), suggesting the role of FphA in cell wall homeostasis (6). Deletion of *fphB* and deletion of both genes had no effect on cell wall homeostasis. At 25 µg/mL Congo red, the inhibitory effect of light on radial growth was observed in all strains (Fig. 3B). None of the constructed deletion strains were more sensitive or resistant to osmotic or oxidative stress (Fig. S4).

### *A. fumigatus* phytochrome-deletion strains show increased pathogenicity

**Fig 4:**
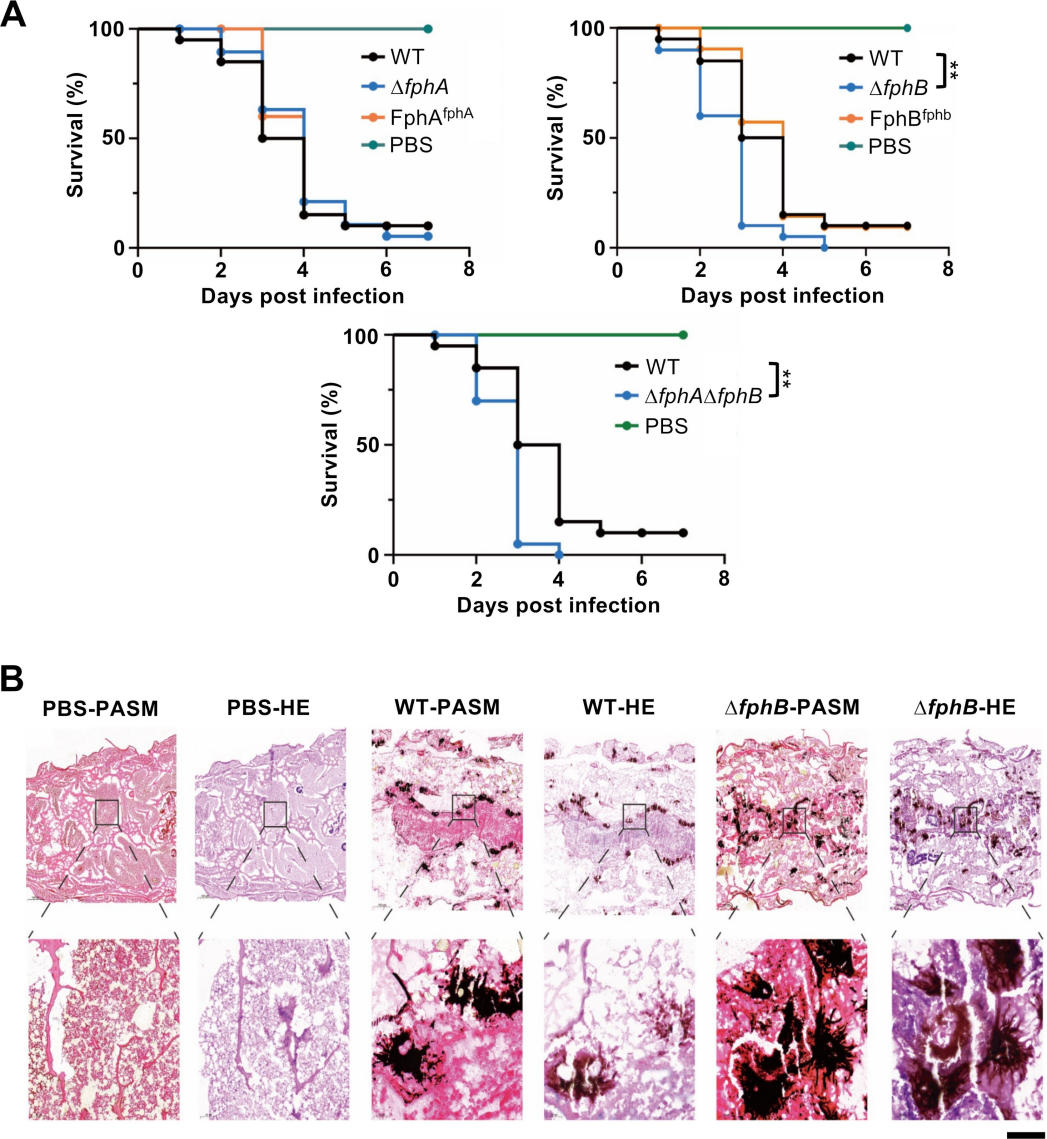
*A. fumigatus* phytochrome B attenuates pathogenicity. (**A**) Survival curves of *G. mellonella* larvae infected with the indicated *A. fumigatus* strains. PBS-injected larvae were used as a negative control. The results are shown with 20 larvae per group (*n* = 20). Statistical differences between groups were determined using a log-rank test. **P < 0.01; no labeling indicates “not significant.” (**B**) Sections were stained with hematoxylin and eosin (HE) and periodic acid-Schiff-methenamine silver (PASM) separately for histological staining analysis. The regions indicated with a square were enlarged in the lower row of pictures. Scale bar, 1 mm (upper row), 100 µm (lower row).

In *A. alternata*, FphA not only acts as a light and temperature sensor but also attenuates pathogenicity (23). Since FphB from *A. fumigatus* appears not to function as a light or temperature sensor, it possibly plays a role, perhaps together with FphA, in pathogenicity. To test this hypothesis, we performed infection assays with larvae of *Galleria mellonella* with WT, ∆*fphA*, ∆*fphB,* and the ∆*fphA*∆*fphB* double-mutant strain and monitored larval survival over 7 days (Fig. 4A). PBS buffer served as control. No significant difference between WT and the *fphA*-deletion strain was observed. Interestingly, larvae infected with the *fphB*-deletion strain or the double-mutant ∆*fphA*∆*fphB* showed significantly reduced survival times. After 5 days, the infection with the ∆*fphB* or after 4 days ∆*fphA*∆*fphB* strains led to death of 100% of the larvae, whereas larvae infected with WT or the *fphA-*deletion strain showed about 20% survival after 7 days. To monitor the fungal burden in the infected larvae, larvae were sectioned 36 h post-infection and the tissue was stained with hematoxylin and eosin (HE). Hyphal cell walls were stained with periodic acid-Schiff-methenamine silver (PASM) and appear therefore dark colored.

### *A. fumigatus* FphB represses genes associated with mycotoxin biosynthesis

**Fig 5:**
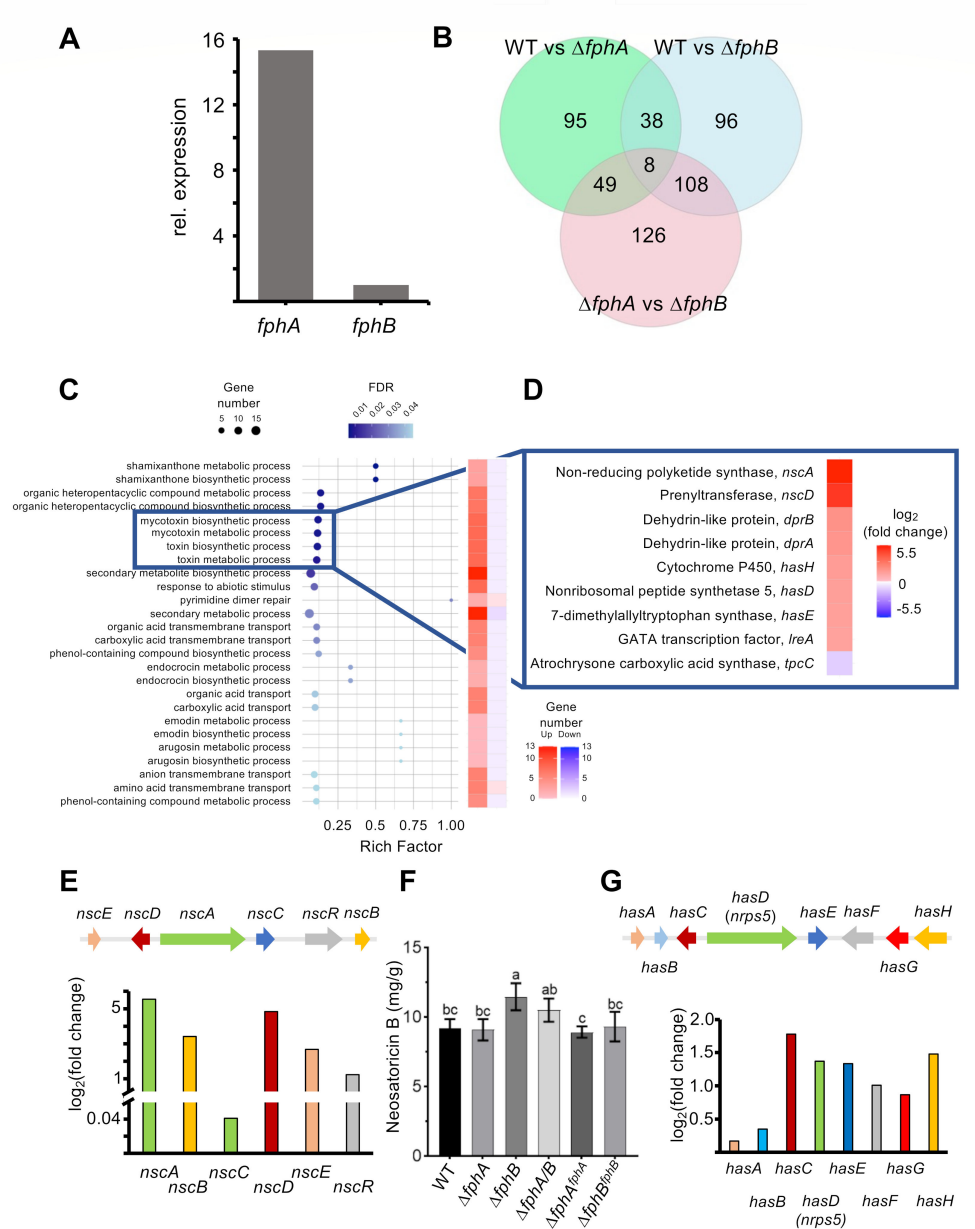
*A. fumigatus* FphB represses two gene clusters responsible for mycotoxin production. (**A**) Relative expression of *fphA* and *fphB* in *A. fumigatus* WT static cultures (normalized to *fphB*). (**B**) Venn diagram of DEGs identified by RNA-seq. (**C**) Enrichment of the subcategories of the GO term “biological processes.” (**D**) Regulation of the individual genes of the subcategories “toxin/mycotoxin biosynthetic/metabolic process.” The subcategories “toxin/mycotoxin biosynthetic/metabolic process” are significantly enriched in *fphB*-deletion strain compared to wild-type. Diagrams in panels **C** and **D** were prepared in R environment using the *ggplot2* package. (**E**) Scheme of the *nsc*-gene cluster and the expression of the individual genes identified by RNAseq. (**F**) Quantification of neosartoricin B. Strains were grown in liquid minimal medium at 37°C in the dark for 48  h under static conditions. Neosartoricin B was extracted from liquid cultures of *A. fumigatus* strains and analyzed by LC-MS/MS. Quantification was normalized to the fresh weight of harvested mycelia. Different letters indicate significant differences at *P* < 0.05 according to one-way ANOVA and *post hoc* Duncan’s test. *n* = 3. (**G**) Scheme of the *has*-gene cluster and the expression of the individual genes identified by RNA-seq.

After the analysis of the role of FphA/B in pathogenicity, we aimed at analyzing effects of the deletion of phytochromes on the transcriptional level. First, we compared the expressions of *fphA* and *fphB* in wild-type when grown in static cultures to mimic the low oxygen environmental conditions during host infection and found that the *fphA* transcript is much more abundant than the *fphB* transcript (Fig. 5A). Under these conditions, about 9,200 genes were identified in an RNA-seq analysis by mapping the transcripts against the genome of *A. fumigatus*. A total of 190 genes were differentially expressed in the *fphA*-deletion strain compared to WT; 92 genes were upregulated, and 98 genes were downregulated (Fig. 5B). Deletion of *fphB* caused differential expression of 250 genes, of which 194 were upregulated and 56 downregulated (Fig. 5B). These 250 genes were further analyzed. Enriched GO terms with a false discovery rate (FDR) below 0.05 are listed in Fig. 5C and D. The rich factor is represented by the dot plot, and the heatmap plot on the right displays the ratio of up and downregulated genes of the respective GO term (Fig. 5D).

The *fphB-*related GO terms Mycotoxin metabolic/biosynthetic process (GO:0043385 and GO:0043386) and toxin biosynthetic/metabolic process (GO:0009403 and GO:0009404) appeared to make sense with regards to the proposed role of phytochrome in pathogenicity. The most upregulated genes are the nonreducing polyketide synthase *nscA* and the prenyltransferase *nscD*, which belong to the *nsc/fcc*-gene cluster (Fig. 5C and D). The cluster encodes the genes for neosartoricin/fumicycline biosynthesis. This prenylated polyphenol polyketide appeared after interaction of *A. fumigatus* with *Streptococcus rapamycinicus* and was discovered at the same time in a genome-mining approach where a cluster-specific transcription factor was activated to induce the other cluster genes (24, 25). The *nsc/fcc-*gene cluster consists of *nsc/fccA-E* and *nsc/fccR* (Fig. 5E). NscR is a C6 transcription factor that regulates the expression of *nscA-E* [log_2_(fold change) = 1.25 in our experiment]. Activation of the *nsc*-gene cluster led to the production of neosartoricin/fumicycline B, which showed antiproliferative activity on murine T-cells (24, 25). The biosynthesis starts with the assembly of the decaketide backbone by the polyketide synthase Nsc/FccA [log_2_(fold change) = 5.54]. Release of the decaketide backbone is accomplished by the metallo-β-lactamase domain protein Nsc/FccB [log_2_(fold change) = 3.42]. The FAD-dependent monooxygenase Nsc/FccC [log_2_(fold change) = 0.04] catalyzes the hydroxylation of the backbone. The prenylation by the dimethylallyl tryptophan synthase Nsc/FccD [log_2_(fold change) = 4.84] leads to neosartoricin B/fumicycline B. The significance of the transcriptional upregulation of many *nsc* cluster genes was further corroborated by analyzing the production of neosartoricin B (Fig. 5F). All strains produced only low amounts of the compound, but the *fphB*-deletion and the *fphA/B* double-deletion strain produced significantly more neosartoricin B than the other strains tested (Fig. 5F). Of course, this analysis has the limitation that the data were obtained from static *A. fumigatus* cultures rather than from infected animals.

The *has*-gene cluster consists of *hasA-H*, including the C6 transcription factor coding genes *hasA* [log_2_(fold change) = 0.17] and *hasF* [log_2_(fold change) = 1.01] (Fig. 5G). The backbone is assembled by the nonribosomal peptide synthase (NRPS) HasD [log_2_(fold change) = 1.37] and further prenylated by the dimethylallyl tryptophan synthase HasE [log_2_(fold change) = 1.34]. The prenylated backbone is hydroxylated by cytochrome P450 HasH [log_2_(fold change) = 1.48] and methylated by the O-methyltransferase HasC [log_2_(fold change) = 1.78]. The prenyl side chain is transferred to a methylbutadienyl side chain by the FAD-binding protein HasG [log_2_(fold change) = 0.87] and the product complexed with Fe(III) to hexadehydrosastechrome (HAS). In addition, the *has*-gene cluster contains a transporter coding gene, *hasB* [log_2_(fold change) = 0.35]. Activation of the gene cluster enhanced virulence (26).

In addition to the *nsc*- and the *has*-gene clusters, the *dpr*-gene cluster (*dprA* and *dprB*) and the GATA transcription factor *lreA* were upregulated. Both have no role in *A. fumigatus* virulence (19, 27). The only downregulated gene was the atrochrysone carboxylic acid synthase *tpcC,* which is involved in the biosynthesis of a conidial secondary metabolite (28).

### Both *A. fumigatus* phytochromes interact in nuclei and the cytoplasm

**Fig 6:**
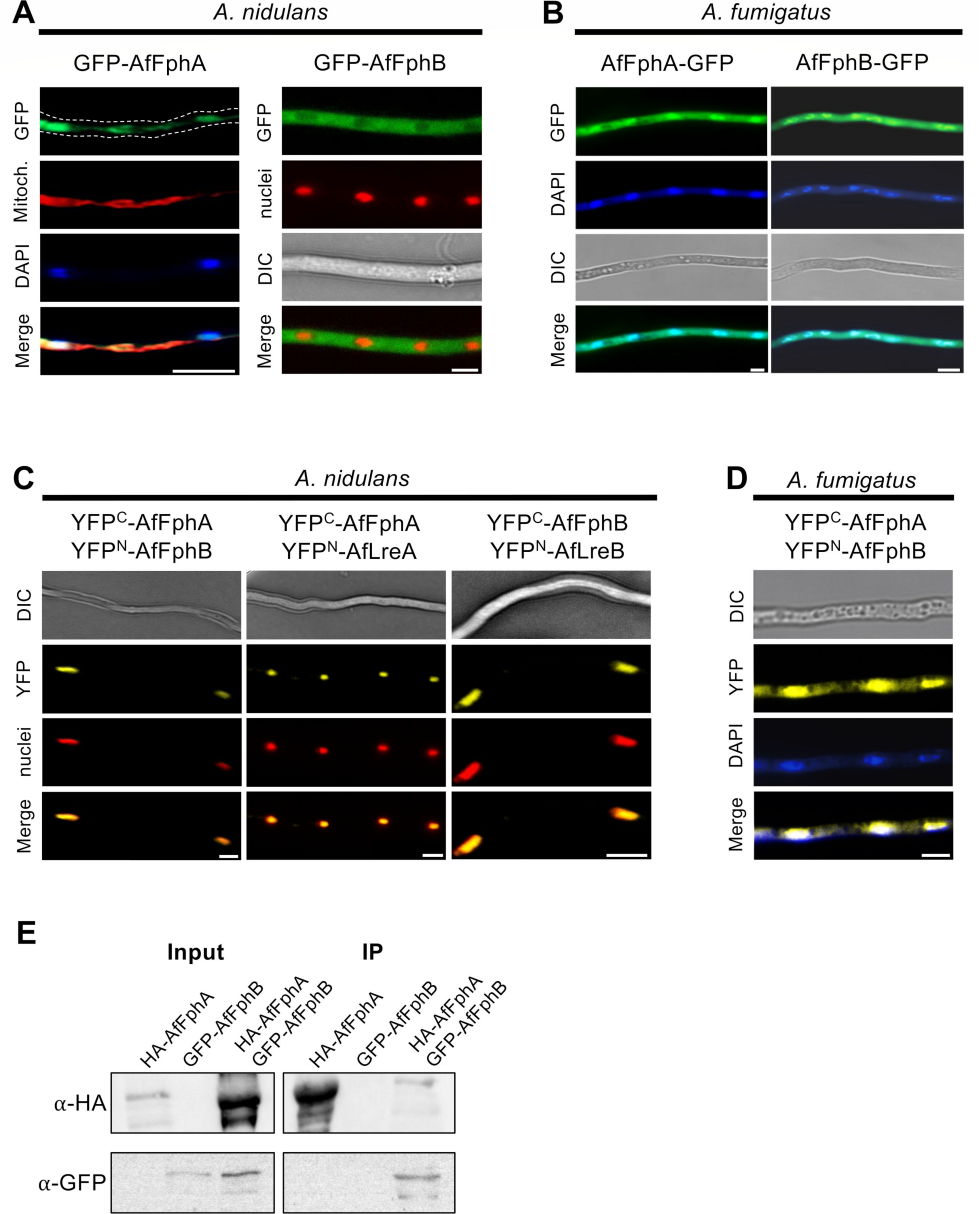
Both phytochromes function and interact in the cytoplasm and nuclei. (**A**) Fluorescence microscopy images of GFP-AfFphA (left) and GFP-AfFphB (right) in *A. nidulans*. AfFphA localizes at the mitochondria and nuclei. AfFphB is located in the cytoplasm. Mitochondria were visualized by MitoTracker staining and nuclei through expression of DsRed fused to the nuclear localization signal domain of StuA (29). (**B**) Fluorescence microscopy images of AfFphA-GFP (left) and AfFphB-GFP (right) in *A. fumigatus*. Both proteins localize in the cytoplasm and nuclei. (**C**) Bimolecular fluorescence complementation of YFP^C^-AfFphA and YFP^N^-AfFphB, YFP^C^-AfFphA and YFP^N^-AfLreA, and YFP^C^-AfFphB and YFP^N^-AfLreB in *A. nidulans* in the dark. Nuclei were stained as in panel **A**. (**D**) Bimolecular fluorescence complementation of YFP^C^-AfFphA and YFP^N^-AfFphB in *A. fumigatus*. Both proteins interact in the cytoplasm and nuclei. Picture taken in light. (**E**) Co-immunoprecipitation of HA-AfFphA and GFP-AfFphB. Precipitation was performed using αHA-agarose beads.

*A. nidulans* phytochrome has cytoplasmic and nuclear functions. Therefore, we tested whether AfFphA and AfFphB reside in the cytoplasm, the nucleus, or in both compartments. Both proteins were N-terminally tagged with GFP and expressed under the control of the inducible *alcA* promoter in *A. nidulans* and in *A. fumigatus*. In *A. nidulans,* AfFphA localized in the cytoplasm at the mitochondria and in nuclei, whereas AfFphB was detected in the cytoplasm (Fig. 6A; Fig. S5). In *A. fumigatus,* both AfFphA and AfFphB were observed in the cytoplasm and in nuclei (Fig. 6B).

In *A. nidulans*, AnFphA forms homodimers and interacts with other proteins of the light-sensing machinery (13). Therefore, we tested if AfFphA and AfFphB could interact at the protein level. AfFphA was tagged with the C-terminal part, AfFphB with the N-terminal part of YFP, and co-expressed in *A. nidulans* or in *A. fumigatus*. A signal was detected in the cytoplasm and in nuclei (Fig. 6C). AfFphA also interacted with AfFphA and AfFphB with AfFphB in *A. nidulans,* suggesting the presence of a light-sensing protein complex. Whether all proteins interact always at the same time or whether transient interactions were “frozen” because of the irreversibility of the split YFP interaction cannot be decided from the experiments. In *A. fumigatus*, the AfFphA/B interaction took place mainly in nuclei, and only a small fraction was observed in the cytoplasm (Fig. 6D). Co-immunoprecipitation of HA-tagged AfFphA and GFP-tagged AfFphB in *A. nidulans* confirmed the interaction observed in the BiFC experiment (Fig. 6E).

### Interaction of FphA and FphB leads to accumulation of SakA (HogA) in the nucleus

FphA in *A. nidulans* functions in the nucleus, where it is involved in chromatin remodeling (15). In the cytoplasm of *A. nidulans*, FphA is activated by red light and in turn activates the SakA (HogA) pathway. The activation results in accumulation of phosphorylated SakA in nuclei where it controls the transcription factor AtfA (14).

To investigate the role of both phytochromes of *A. fumigatus*, the *A. nidulans* strains expressing one or the two *A. fumigatus* phytochromes (*AffphA* [SKL2], *AffphB* [SKL3], and *AffphA* and *AffphB* [SKL6.10]) as well as a strain expressing the *A. nidulans* FphA protein and the *A. fumigatus* FphB protein (*AnfphA* and *AffphB* [SKL14]) were transformed with plasmids encoding GFP-SakA and DsRed as nuclear label {pKL71 [*alcA(p)::GFP::sakA*] and pJW18 [*alcA(p)::stuA(NLS)::DsRed*]}). An *AnfphA*-deletion strain expressing *GFP::sakA* served as the negative control.

**Fig 7:**
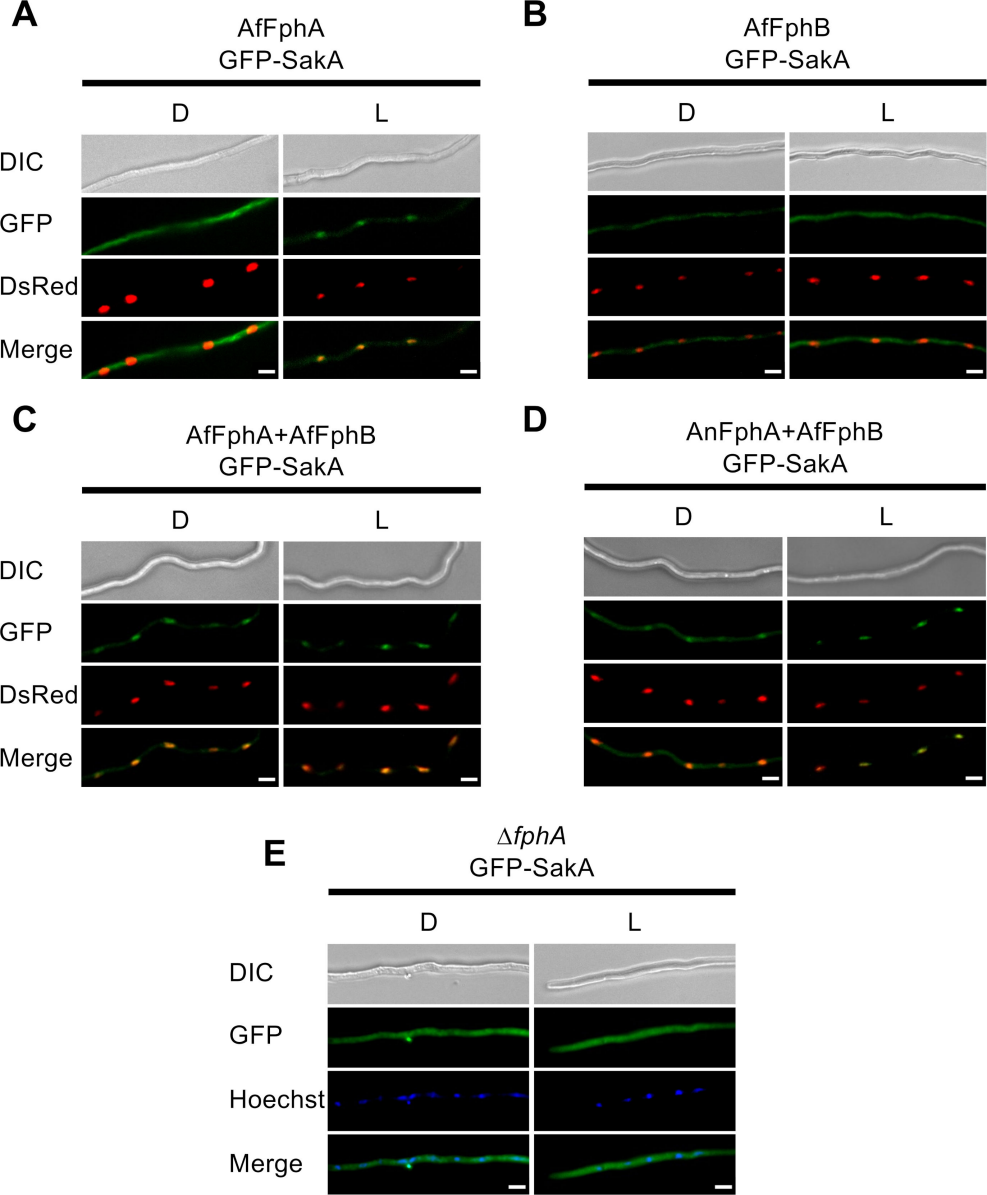
AfFphA activates the SakA signaling pathway in light, and AfFphA and AfFphB together activate constitutively the SakA signaling pathway in the dark. (**A**) Localization of GFP-SakA in *A. nidulans* ∆*AnfphA* expressing *AffphA* in the dark (D) and in light (L). In the dark, GFP-SakA localizes in the cytoplasm. In light, the signal accumulates in the nuclei. (**B**) Localization of GFP-SakA in *A. nidulans* ∆*AnfphA* expressing *AffphB* in the dark (D) and in light (L). GFP-SakA localizes in the cytoplasm in the dark as well as in light. (**C, D**) Localization of GFP-SakA in *A. nidulans* ∆*AnfphA* expressing AfFphA and AfFphB (**C**) or AnFphA and AfFphB (**D**). In both cases, SakA accumulates in the nuclei in the dark as well as in light. (**E**) Localization of GFP-SakA in *A. nidulans* ∆*fphA* as negative control.

The strain only expressing *AffphA* showed a GFP-SakA signal in the cytoplasm when kept in the dark. Exposed to light, the GFP signal was observed in nuclei (Fig. 7A). When expressing *AffphB* alone, GFP-SakA remained in the cytoplasm, independent of light (Fig. 7B). Interestingly, the combination of either AfFphA or AnFphA with AfFphB caused an accumulation of GFP-SakA in nuclei, independent of light (Fig. 7C and D). Expressing *GFP::sakA* in a ∆*AnfphA* background resulted in a failure of SakA to shuttle into nuclei in light (Fig. 7E).

## DISCUSSION

Fungal phytochromes have been functionally characterized primarily in *Aspergillus nidulans* and *Alternaria alternata*. In the opportunistic human pathogen *A. fumigatus*, two putative phytochromes were identified, though only one had previously been studied via gene deletion. In this study, we investigated both proteins and found that only one, FphA, is photoactive, functioning in light and temperature sensing similar to its ortholog in *A. nidulans* (5, 11, 16). The second phytochrome, FphB, lacks photoactivity, likely acting as a hybrid histidine kinase. FphB physically interacts with FphA in both the cytoplasm and nuclei, suggesting a regulatory relationship. While FphB does not influence light or temperature sensing, its deletion increases pathogenicity in the *Galleria mellonella* model, indicating a role in virulence attenuation. Overexpression of both *A. fumigatus* FphA and FphB in *A. nidulans* led to constitutive nuclear localization of the MAP kinase SakA (HogA), independent of light, suggesting hyperactivation of FphA by FphB. In *A. fumigatus*, this effect likely does not occur due to FphB’s significantly lower expression—approximately 15-fold less than FphA (Fig. 5A). Consequently, only a small fraction of FphA would be affected by FphB under native conditions, explaining why *fphB* deletion does not alter FphA-mediated light responses.

FphA appears to activate the HOG pathway by dephosphorylating the phosphotransfer protein YpdA, and *in vitro* data suggest its response regulator domain can accept a phosphate from YpdA (30). While the precise mechanism by which light influences this phosphotransfer remains unclear, it is plausible that light induces conformational changes in FphA, modulating its kinase activity and exposure of the response regulator domain—potentially the site of FphB-mediated regulation.

While the physical interaction between FphA and FphB may explain how FphB influences the light- and temperature-sensing functions of FphA, this interaction alone is unlikely to account for the observed increase in pathogenicity upon *fphB* deletion, especially since FphA itself does not appear to contribute to virulence. One possible explanation is that FphB interacts not only with FphA but also with other hybrid histidine kinases in *A. fumigatus*. The genome encodes thirteen such signaling modules, many of which are implicated in stress response pathways. Notably, members of family III and X have been proposed to play potential roles in pathogenicity (21). Thus, FphB may act as a broader regulatory hub within the histidine kinase network, modulating multiple pathways, including those relevant to fungal virulence.

An alternative explanation for the role of FphB in attenuating virulence may lie in its nuclear functions, potentially in cooperation with FphA. In *A. nidulans*, FphA contributes to chromatin remodeling and interacts with key transcriptional regulators, including the velvet protein VeA and the WC-2 ortholog LreB (31). It is conceivable that FphB, possibly in complex with FphA, modulates the activity of such regulators. VeA, in particular, governs morphogenesis and secondary metabolism, and its activity is controlled by a phosphorylation code (32–35). Misregulation of VeA or similar factors may explain the developmental phenotype observed when *A. fumigatus* FphB was co-expressed with *A. nidulans* FphA. In *A. fumigatus*, VeA regulates hundreds of genes, including those involved in secondary metabolite biosynthesis (36, 37)*.* Our transcriptome analysis showed that deletion of *fphB* slightly activated two secondary metabolite gene clusters*—nsc* and *has*—leading to increased production of neosartoricin B (fumicycline B) and likely also to hexadehydroastechrome, both linked to pathogenicity. Neosartoricin B suppresses host immune responses, and its transcriptional activator *nscR* is strongly upregulated in infected lung tissue (25, 38). Hexadehydroastechrome has also been associated with hypervirulence (26). Within the *has* cluster, although expression of the C6 transcription factor *hasA* was largely unchanged upon *fphB* deletion, target biosynthetic genes were upregulated, suggesting that FphB may regulate HasA post-translationally. As a hybrid histidine kinase, FphB could modulate HasA activity via phosphorylation.

While our data reveal a modest increase in metabolite levels and gene expression, it is important to note that these results were obtained from static cultures intended to simulate host-like conditions. However, true *in vivo* environments—such as those within *G. mellonella* or murine models—likely provide additional regulatory signals that further modulate these pathways. Investigating these in-host dynamics will be crucial for a deeper understanding of the role of FphB in pathogenicity.

In summary, the hybrid histidine kinase FphB emerges as a potential novel virulence factor in *A. fumigatus*. Its interaction with FphA and the apparent importance of its expression level for regulatory function highlight the need to elucidate the upstream regulatory circuits controlling *fphB* expression. Understanding these mechanisms will be key to clarifying FphB’s role in fungal pathogenicity.

## MATERIALS AND METHODS

### Strains, plasmids, and culture conditions

Strains used in this study are listed in Table 1. *A. nidulans* strains were constructed according to standard procedures (39), and supplemented minimal medium (MM) was prepared as described previously (40, 41). *E. coli* TOP10 was used for plasmid amplification. Heterologous expression of recombinant proteins was carried out using *E. coli* BL21 (DE3).

**TABLE 1:** Fungal and bacterial strains used in this study

Strain	Genotype	Source
*A. nidulans*		
FGSC A4	Wild type	Fungal Genetics Stock Center
SJP1	*pyrG89;* ∆*argB::trpCDB; pyroA4;* ∆*fphA::argB; veA+*	(13)
SJP22.1	SJP1 re-complemented with A*nfphA(p)::AnfphA; pyr4*	(13)
SKV16	∆*argB;* ∆*pyroA4, veA+*	K. Vienken (unpublished data)
SKV103	*pyrG89; pyroA4; veA*+	(42)
SKL2	*AnfphA(p)::AffphA;* ∆*argB::trpCDB; pyroA4;* ∆*fphA::argB; veA+* (SJP1 transformed with pKL32)	This study
SKL3	*AnfphA(p)::AffphB;* ∆*argB::trpCDB; pyroA4;* ∆*fphA::argB; veA+* (SJP1 transformed with pKL33)	This study
SKL4	*alcA(p)::GFP::AffphA;); pyroA4; veA+* (SKV103 transformed with pKL60)	This study
SKL6.10	*AnfphA(p)::AffphA; AnfphA(p)::AffphB;* ∆*argB::trpCDB;* ∆*fphA::argB; pyroA; veA+* (SKL2 transformed with pKL48)	This study
SKL14	*AnfphA(p)::AffphB, pyroA4; veA+* (WT SKV103 transformed with pKL33)	This study
SKL15	*alcA(p)::DsRed(T4)::stuA(NLS); pyroA4; veA+*	This study
SKL16	*alcA(p)::GFP::AffphA; alcA(p)::DsRed(T4)::stuA(NLS); pyroA4; veA+* (SKV103 transformed with pKL60 and pJW18)	This study
SKL17	*alcA(p)::GFP::AffphB; alcA(p)::DsRed(T4)::stuA(NLS); pyrG89; veA+* (SKV103 transformed with pKL62 and pJW18)	This study
SKL18	*alcA(p)::YFP^N^::AffphB; alcA(p)::YAP^C^::AffphA; alcA(p)::DsRed(T4)::stuA(NLS); veA+* (SKV103 transformed with pKL65, pKL66 and pJW18)	This study
SKL23	*AnfphA(p)::AffphA; AnfphA(p)::AffphB; alcA(p)::GFP::sakA; alcA(p)::DsRed(T4)::stuA(NLS);* ∆*argB::trpCDB;* ∆*fphA::argB; pyroA; veA+* (SJP1 transformed with pKL32, pKL48, pKL71 and pJW18)	This study
SKL24	*AnfphA(p)::AffphB; alcA(p)::GFP::sakA; alcA(p)::DsRed(T4)::stuA(NLS); pyroA; veA+* (SKL15 transformed with pKL48 and pKL71)	This study
SKL25	*alcA(p)::GFP::AffphB; alcA(p)::3×HA::AffphA;* ∆*argB::trpCDB;* ∆*fphA::argB; veA+* (SJP1 transformed with pKL62 and pKL74)	This study
SKL26	*alcA(p)::3×HA::AffphA;* ∆*argB::trpCDB; pyroA4;* ∆*fphA::argB; veA+* (SJP1 transformed with pKL74)	This study
SKL29	*alcA(p)::GFP::sakA; pyrG89; ∆argB::trpCDB;* ∆*fphA::argB; veA+* (SJP1 transformed with pKL76)	This study
SKL30	*AnfphA(p)::AffphA; alcA(p)::gfp::sakA; alcA(p)::DsRed(T4)::stuA(NLS);* ∆*argB::trpCDB;* ∆*fphA::argB; veA+* (SKL2 transformed with pKL76 and pJW18)	This study
SKL31	*AnfphA(p)::AffphB; alcA(p)::gfp::sakA; alcA(p)::DsRed(T4)::stuA(NLS);* ∆*argB::trpCDB;* ∆*fphA::argB; veA+* (SKL3 transformed with pKL76 and pJW18)	This study
*A. fumigatus*		
CEA17 ∆akuB^KU80^	∆*ku80, pyrG^+^*	(43)
KU80	∆*ku80*	This study
*∆fphA*	∆*ku80,fphA::hph*	This study
*∆fphB*	∆*ku80,fphB::hph*	This study
*∆fphA∆fphB*	∆*ku80,fphA::hph;* ∆*fphB::phel*	This study
OE *fphA*	∆*ku80; gpd(p)::fphA::hph*	This study
OE *fphB*	∆*ku80; gpd(p)::fphB::hph*	This study
AF1160	∆*ku80,pyrG*	This study
FphA-GFP	∆*ku80,pyrG; fphA::GFP::pyr4*	This study
FphB-GFP	∆*ku80,pyrG; fphB::GFP::pyr4*	This study
FphA-YFP^C^ FphB-YFP^N^	*alcA(p)::YFP^C^::AffphA::pyr4; alcA(p)::YFP^N^::AffphB::hph*	This study

Enzymes as well as DNA and protein markers were provided by New England Biolabs (Frankfurt, Germany), Thermo Scientific (Waltham, Massachusetts, USA), and Fermentas (St-Leon-Rot, France). All plasmids used in this study are listed in Table 2. Respective oligonucleotides used for amplification and cloning are shown in Table 3. Plasmids were constructed either by Gibson assembly (NEBuilder HiFi DNA Assembly Cloning Kit, NEB) or T4 ligation. Plasmids for *A. nidulans* transformation derived from pMCB17apx (44). For protein expression in *E. coli*, codon-optimized synthetic versions of *A. fumigatus fphA* and *fphB* were cloned in the pASK-iba3 vector (IBA Lifesciences, Göttingen, Germany). The Q5 Site-Directed Mutagenesis Kit (NEB) was used to obtain *AffphB-M203C*.

**TABLE 2:** Plasmids used in this study

Plasmid	Feature	Source
pKL09	*tet(p)::AffphBs::strep-tag; ampR* (codon optimized *AffphB* in pASK-iba3 backbone)	This study
pKL10	*tet(p)::AffphAs-npgp::strep-tag; ampR* (first 742 amino acids of codon optimized *AffphA* photosensory module in pASK-iba3 backbone)	This study
pACYCDuet-1_bphO	*bphO* from *P. aeruginosa* (PA4116) in *BglII* + *XhoI; lac(p); CmR*	A. Ali, Karlsruhe, Germany
pJW18	*alcA(p)::DsRed(T4)::stuA(NLS); argB*	(29)
pMCB17apx	*alcA(p)::GFP; pyr4; ampR*	(44)
pKL32	*AnfphA(p)::AffphA; pyr4; ampR* (pMCB17apx derivative)	This study
pKL33	*AnfphA(p)::AffphB; pyr4; ampR* (pMCB17apx derivative)	This study
pKL48	*AnfphA(p)::AffphB; pyroA; ampR* (pMCB17apx derivative)	This study
pKL60	*alcA(p)::GFP::AffphA; pyr4; ampR* (pMCB17apx backbone)	This study
pKL62	*alcA(p)::GFP::AffphB; pyroA; ampR* (pMCB17apx backbone; *pyr4* replaced by *pyroA*)	This study
pKL65	*alcA(p)::YFP^N^::AffphB; pyroA; ampR* (pMCB17apx backbone; *pyr4* replaced by *pyroA* and *GFP* replaced by YFP*^N^*)	This study
pKL66	*alcA(p)::YFP^C^::AffphA; pyr4; ampR* (pMCB17apx backbone; *GFP* replaced by YFP*^C^*)	This study
pKL71	*alcA(p)::GFP::sakA; pyr4; ampR* (pMCB17apx backbone)	This study
pKL74	*alcA(p)::3xHA::AffphA; pyr4; ampR* (pMCB17apx backbone; *GFP* replaced by *3*×*HA)*	This study
pKL76	*alcA(p)::GFP::sakA; pyroA; ampR* (pMCB17apx backbone; *pyr4* replaced by *pyroA)*	This study
AfFphB-YFP^N^	*alcA(p)::YFP^N^::AffphB; hph; ampR* (pAN7-1apx backbone)	This study

**TABLE 3:** Oligonucleotides used in this study

Name	Sequence (5′−3′)	Description
AffphA/B fwd	GTGAAATGAATAGTTCGACAAAAATCTAGAAATAATTTTGTTTAACTTTAAGAAGG	Heterologous expression of *AffphA-NPGP/AffphB*
AffphA-NPGP rv	AACTTTAATGAATTTCCCATAAACCAGAC	Heterologous expression of *AffphA-NPGP*
AffphB rv	GGGTGGCTCCAAGCGCTGAGACCATGGTCGCTTTCACCG	Heterologous expression of *AffphB*
AnfphA(p) + EcoRI fw	GTAAAACGACGGCCAGTgaattcCTTGGTTGTTGCGGCAATGT	Fusion of *AnfphA(P)* and *AffphA/AffphB*
AnfphA(p) rv	GCTCGACAAGGAAGAGCAAG	Fusion of *AnfphA(P)* and *AffphA/AffphB*
AffphA + AnFphA(p) fw	CTTGCTCTTCCTTGTCGAGCATGGCGTCAAGAGCCAATGCC	Fusion of *AnfphA(P)* and *AffphA/AffphB*
AffphA + BamHI rv	CAGGTCGACTCTAGAGGATCCTCAGAGCTCCCCATGGTGTT	Fusion of *AnfphA(P)* and *AffphA/AffphB*
AffphB + AnFphA(p) fw	CTTGCTCTTCCTTGTCGAGCATGAGAAGCAACAGAATATTG	Fusion of *AnfphA(P)* and *AffphA/AffphB*
AffphB + BamHI rv	CAGGTCGACTCTAGAGGATCCTTAGCTCTCACCTCCCTTATCG	Fusion of *AnfphA(P)* and *AffphA/AffphB*
AffphA + AscI fw	CGCTggcgcgccAGCGTCAAGAGCCAATGCCT	*GFP-AffphA*
AffphA + PacI rv	TCTAGAGGATCCttaattaaTCAGAGCTCCCCATGGTGTT	*GFP-AffphA*
AffphB + AscI fw	CGCTggcgcgccAAGAAGCAACAGAATATTGAGATCTCGAAAAC	*GFP-AffphB*
AffphB + PacI rv	TCTAGAGGATCCttaattaaTTAGCTCTCACCTCCCTTATCG	*GFP-AffphB*
sakA + AscI fw	ATGGATGAACTATACAAAggcgcgccATGGCGGAATTTGTACGTGCC	*GFP-sakA*
sakA + PacI rv	CTCAACCAGCAAGGTTTCCAATAAttaattaaGGATCCTCTAGAGTC	*GFP-sakA*
RT_AN_ccgA_100 bp fw	CGCTTCCCTCACTTCTCGT	RT-qPCR *ccgA*
RT_AN_ccgA_100 bp rv	TTCTTAGCGGCCTCCTTGTG	RT-qPCR *ccgA*
RT_AN_h2b_100 bp fw	GAAGAAGCGCGGAAAGACC	RT-qPCR *h2b*
RT_AN_h2b_100 bp rv	TAGACATAGCACGGGTGGAG	RT-qPCR *h2b*
RT_AN_ccgB_100 bp fw	ATAACGCCGACCTGACTACG	RT-qPCR *ccgB*
RT_AN_ccgB_100 bp rv	TTGGCGGCTTCCTTGTAAAC	RT-qPCR *ccgB*
Oligonucleotides used for RTqPCR		
RT_AN_h2b_100 bp fw	GAAGAAGCGCGGAAAGACC	
RT_AN_h2b_100 bp rv	TAGACATAGCACGGGTGGAG	
RT_AN_ccgA_100 bp fw	CGCTTCCCTCACTTCTCGT	
RT_AN_ccgA_100 bp rv	TTCTTAGCGGCCTCCTTGTG	
RT_AN_ccgB_100 bp fw	ATAACGCCGACCTGACTACG	
RT_AN_ccgB_100 bp rv	TTGGCGGCTTCCTTGTAAAC	
Oligonucleotides used for constructing fphA-deletion and *fphB*-deletion strains in *A. fumigatus*		
fphA P1	CAACTACAAATCCCATCTCCCC	
fphA P2	TTCCGTAATGACCCCGATGA	
fphA-P3-hph	CGGCGGATTTTAGGCTCAAGGTAAAACAAAAGCGGCGACC	
fphA-P4-hph	GTTGCCTAGTGAATGCTCCGATCACCATCGACACCTCACC	
fphA P5	GGATGAGGTATGATGGCGGA	
fphA P6	TCTCTTCCCCATTCCATGCC	
fphA-self-F	AGCACCCTTGAGGCTTTGTT	
fphA-self-R	TGGCCTGGAATACATCACGG	
fphB-P1	GGACGGAAAGCCTCTCATCA	
fphB-P2	CCCAAAGCAGTGATGTTCCT	
fphB-P3-hph	CGGCGGATTTTAGGCTCAAGTTCCGATGCTGTCAGAAGGA	
fphB-P4-hph	GTTGCCTAGTGAATGCTCCGTCGGTATGAGACATAGGCGG	
fphB-P5	TTACTCCTCGGTCTCAGGGA	
fphB-P6	GCTTACGCTCAGGGAACATA	
fphB-self-F	TCAGATGCAGGGCCCTTTTA	
fphB-self-R	TCTCGGTATAGTCGGGCAAC	
Oligonucleotides used for constructing *∆fphA∆fphB* double-mutant in *A. fumigatus*		
fphB-phel-P3	TAATCAATTGCCCGTCTGTCATTCCGATGCTGTCAGAAGGA	
fphB-phel-P4	GCTTACATTCACGCCCTCCTTCGGTATGAGACATAGGCGG	
Oligonucleotides used for constructing overexpression of *fphA* and *fphB* strains in *A. fumigatus*		
GPD-fphA-hph-F	GGGCTGCAGGAATTCGATATCATGGCGTCAAGAGCCAATGC	
GPD-fphA-hph-R	GGTATCGATAAGCTTGATATCGTTCATTCCTCCCAACTGTCTGA	
GPD-fphB-hph-F	GGCTGCAGGAATTCGATATCATGAGAAGCAACAG AATATTGAGATCTC	
GPD-fphB-hph-R	GGTATCGATAAGCTTGATATCTTGGTTCTACTGGACCTGGCG	
GPD-F-yan	ACAAGCTGTGACCGTCTCC	
GPD-fphA-hph-F	GGGCTGCAGGAATTCGATATCATGGCGTCAAGAGCCAATGC	
GPD-fphA-hph-R	GGTATCGATAAGCTTGATATCGTTCATTCCTCCCAACTGTCTGA	
GPD-fphB-hph-F	GGGCTGCAGGAATTCGATATCATGAGAAGCAACA GAATATTGAGATCTC	
GPD-fphB-hph-R	GGTATCGATAAGCTTGATATCTTGGTTCTACTGGACCTGGCG	
Oligonucleotides used for constructing FphA-GFP and FphB-GFP strains in *A. fumigatus*		
fphA-GFP-P1	AGGCCAAGCGTAAGAGCATT	
fphA-GFP-P2	GCGTTGGTTGCTCGAATTGT	
fphA-GFP-P3	CCAGCGCCTGCACCAGCTCCGAGAGCTCCCCATGG	
fphA-GFP-P4	CATCAGTGCCTCCTCTCAGACAGCGCATCACC ATCGACACCTC	
fphA-GFP-P5	ATGATGGCAGCGTATTCCGT	
fphA-GFP-P6	TTTCTCGTCACCACCCTTGG	
fphA-GFP-yan-F	AGATCGCTTTGGAAGGCTCC	
fphA-GFP-yan-R	GCTTGCGTTTGGCGTCCGTCTTGCG	
fphB-GFP-P1	TGCCCGACTATACCGAGACT	
fphB-GFP-P2	TCCATGACACTGGAACTGGC	
fphB-GFP-P3	CCAGCGCCTGCACCAGCTCCGCTCTCACCTCCC	
fphB-GFP-P4	CATCAGTGCCTCCTCTCAGACAGGAACTTGTGGAACTG	
fphB-GFP-P5	ACGACCCTTATGTGGCGAAG	
fphB-GFP-yan-F	AAGAAGCGAGGCAAAAGGGT	
fphB-GFP-yan-R	CCGCATTCAATATTCCTACTGGGCG	

### Heterologous expression of FphA and FphB from *A. fumigatus* in *E. coli*

Recombinant FphA and FphB from *A. fumigatus* were produced using a C-terminal *Strep-tag E. coli* expression system driven by a *tet* promoter. For *in vivo* assembly of the respective holo-phytochrome, both phytochromes were co-expressed with the bacterial heme oxygenase BphO from *P. aeruginosa.* Cells were grown at 37°C in 1 L LB medium with ampicillin (50 µg/mL), chloramphenicol (30 µg/mL), sorbitol (100 mM), and betaine (2.5 mM) to an OD_600_ of 0.6. Bilin biosynthesis was induced by addition of 250 µM IPTG 1 hour prior to induction of FphA or FphB. FphA or its variants were induced by addition of 0.2 µg/mL anhydrotetracycline, and cells were incubated overnight at 20°C. The bacterial pellet of a 1 L culture was suspended in 20 mL extraction buffer (50 mM Tris-HCl [pH 7.8], 300 mM NaCl, 10% glycerol, 0.05% Tween 20, 2 mM DTT, 1 mM PMSF). The cells were lysed using an EmulsiFlex-C3 high-pressure homogenizer (Avestin, Inc., Canada) at 1,000–1,500 bar, and the cell debris was removed by centrifugation. The supernatant was incubated with 40 µg/mL avidin for 20 minutes on ice. Recombinant proteins were enriched using ӒKTA pure chromatography system (Cytiva, USA) and a StrepTrap HP (5 mL) column. Elution was performed in elution buffer (50 mM Tris-HCl [pH 7.8], 300 mM NaCl, 10% glycerol, 0.05% Tween 20, 2 mM TCEP, 5 mM D-desthiobiotin).

### Bioinformatics

Reference sequences were received from National Center for Biotechnology Information (NCBI). Multiple sequence alignments were done with *clustalW*.

### Spectroscopy

The spectra were recorded in a JASCO V-750 photometer at room temperature. Sample irradiation was carried out using a custom-built irradiation device. To ensure the ground state of the respective phytochrome, samples were kept in the dark or green safety light. Pfr state was established by irradiating the protein sample for 2 minutes with red light (642 nm, 16 µmol/m^2^s). Reversion of the Pfr state back to the Pr state was accomplished by irradiation with far-red light (782 nm, 863 µmol/m^2^s).

### SDS-PAGE and zinc-induced red fluorescence

Zinc ions form fluorescent complexes with biliproteins and peptides that can be excited by UV light (45). To visualize covalent chromophore attachment, samples of purified FphA and FphB, as well as FphB^M203C,^ were subjected to sodium dodecyl sulfate-polyacrylamide gel electrophoresis (SDS-PAGE), as described (46). Zinc acetate (1 mM) was added to Tris-glycine running buffer and Tris-buffers used for preparation of 5% stacking gel and 10% separating gel. After visualization by UV light, the gel was stained in Coomassie brilliant blue staining solution (45% methanol, 10% acetic acid, 45% ddH_2_O, 0.1% Coomassie Brilliant Blue R-250) for 30 minutes and destained in destaining solution (40% methanol, 10% of 100% acetic acid, 50% ddH2O). The Qubit fluorometer was used for protein concentration determination.

### Co-immunoprecipitation and immunodetection

*A. nidulans* strains expressing HA-AfFphA, GFP-AfFphB, or both were cultured in 500 mL minimal medium (0.2% glucose and 2% threonine) at 37°C and 180 rpm for 24 hours. Mycelia were harvested by filtration, washed with 50 mL PBS, and dried between filter towels before freezing in liquid nitrogen. The dried samples were ground in a mortar, and 1.5 mL protein extraction buffer (150 mM NaCl, 100 mM Tris-HCl [pH 8.0], 0.05% Tween 20) containing 1 mM PMSF and a protease inhibitor mix (Pierce Protease Inhibitor Tablets EDTA-free, Thermo Fisher Scientific) was added and incubated on ice for 30 minutes. Samples were clarified by centrifugation twice for 15 minutes at 13,000 rpm and 4°C, and the protein concentrations were determined with a Qubit fluorometer.

For immunoprecipitation, 12 mg total protein of each sample was incubated with 40 µL settled anti-HA agarose resin (Pierce Anti-HA Agarose, Thermo Fisher Scientific, Waltham, MA) for 3 hours at 4°C on a rotary device. The agarose beads were washed five times and incubated 5 minutes at 95°C in 40 µL 2× loading dye to release bound protein. The agarose beads were pelleted by centrifugation, and the supernatants, as well as 300 µg total protein of the protein extracts as input controls, were subjected to SDS-PAGE, as described above.

The separated proteins were transferred to a nitrocellulose membrane by electroblotting (2 h, 100 V, 4°C). Immunodetection was performed first with anti-HA monoclonal antibody (H3663, Sigma-Aldrich, 1:2,000) as primary and anti-mouse IgG (Fab specific)-peroxidase (A2304, Sigma-Aldrich, 1:80,000) as secondary antibody. The membrane was stripped and immunodetection repeated with anti-GFP polyclonal primary (G1544, Sigma-Aldrich, 1:2,000) and anti-rabbit IgG (whole molecule)-peroxidase secondary antibodies (A0545, Sigma-Aldrich, 1:80,000).

### Quantification of conidia

Determination of *A. nidulans* spore number was performed as described with slight modifications (15). In brief, spores (1 × 10^5^) of different strains were inoculated on solid YAG media (5 g yeast extract, 10 g glucose, 15 g agar, 1.0 g uracil, 1.0 g uridine, 10 mM MgSO_4_, 1 mL trace elements) and grown for 3 days at 37°C either in full darkness or illuminated with white light (200 µmol photons/m^2^*s^2^). Spores were harvested in 15 mL dH_2_O and counted in a Neubauer chamber. Three biological replicates were analyzed.

### Microscopy

Localization and interaction studies were done by fluorescence microscopy. To this, fresh conidia of the respective *A. nidulans* strain were inoculated in microscopy medium [0.2% glucose, 2% glycerol, 1 g/L uridine, 1 g/L uracil, 0.1 g/L biotin, 0.1 g/L pyridoxine, 0.1 g/L thiamine, 0.1 g/L riboflavin, 0.1 g/L *p*-aminobenzoic acid, 0.1 g/L nicotinic acid, 6 g/L NaNO_3_, 0.52 g/L KCl, 0.52 g/l MgCl_2_ × 7H_2_O, 1.52 g KH_2_PO_4_, 22 mg/L ZnSO_4_ × 7H_2_O, 11 mg/L H_3_BO_3_, 5 mg/L MnCl_2_ × 4H_2_O, 5 mg/L FeSO_4_ × 7H_2_O, 1.6 mg/L CuSO_4_ × 5H_2_O, 1.1 mg/L (NH_4_)_6_Mo_7_O_24_ × 4H_2_O, 1.6 mg/L CoCl_2_ × 5H_2_O, 50 mg/L Na_4_EDTA] on cover slips and incubated 16 hours at 25°C in the dark. In case of bimolecular fluorescence complementation, samples were analyzed after incubation in the dark. To analyze the activation of SakA, samples were either kept in darkness or irradiated with white light (5 µmol/m^2^s) for 5 minutes prior to fixation with 4% *p*-formaldehyde (50 mM PIPES, 25 mM EGTA, 5 mM MgSO_4_, 5% DMSO, 4% *p*-formaldehyde). Samples were washed with PBS and analyzed on a Zeiss Axio Imager Z1 fluorescence microscope.

Localization and interaction of AfFphA and AfFphB in *A. fumigatus* were analyzed on an Olympus fluorescence microscope. Fresh conidia of the respective strain were inoculated in minimal medium (1 × 10^5^ spores/mL) on cover slips. For localization experiments, samples were incubated at 37°C for 13 h in the dark. The medium was removed, and the samples washed twice with PBS and incubated with DAPI for 10 minutes at room temperature. To analyze the interaction by bimolecular fluorescence microscopy, minimal medium containing glycerol (1%) and L-threonine (11.9 g/L) instead of glucose was inoculated with fresh spores of the respective strain (1 × 10^5^/mL) and incubated on cover slips for 12 h at 37°C in the dark. Samples were moved to 28°C and incubated further for 10 h in the dark. After white light treatment for 1 h at 28°C, the medium was removed and the samples washed twice with PBS. Samples were incubated with DAPI for 10 minutes at room temperature.

### RNA isolation and RT-qPCR

Fresh conidia of the used strains were inoculated on the surface of 10 mL supplemented minimal medium (3 × 10^8^ spores) and incubated in the dark for 18 h at 37°C in petri dishes. Light-dependent gene induction was investigated keeping the samples either in the dark (dark control) or treated for 15 minutes with red light (700 nm, 2 µmol/m^2^s). For temperature-dependent gene induction, mycelia were transferred to pre-warmed minimal medium at either 28°C or 42°C, respectively. Samples were incubated for 8 minutes. After treatment, mycelia were harvested in dim green light and immediately frozen in liquid nitrogen. Frozen mycelia were ground in liquid nitrogen in a mortar, and RNA was extracted using an E.Z.N.A. total DNA/RNA isolation kit (Omega Bio-tek Inc., USA). DNA was digested using the TURBO DNA-free kit (Invitrogen, Thermo Fisher Scientific). Gene expression of three biological and two technical replicates was analyzed by reverse transcriptase-quantitative PCR (SensiFAST SYBR No-ROX One-Step Kit, Bioline). Three biological replicates and technical duplicates were used. Expression levels were normalized to h2b. Primers used are listed in Table 3.

### RNA sequencing

To mimic the low oxygen environmental conditions during host infection, spores of *A. fumigatus* WT, ∆*fphA,* and ∆*fphB* were cultured in liquid static cultures. Therefore, spores of the respective strain were inoculated in minimal medium (10^5^ spores/mL) in petri dishes and incubated for 24 hours at 37°C in the dark without shaking. Three biological replicates were used per strain. RNA extraction, sequencing, and data analysis were performed by Shanghai Personal Biotechnology Co. ,Ltd.

After RNA extraction, the quality of total RNA was verified using the Agilent 2100 Bioanalyzer. polyA-mRNA was enriched by oligo(dT) magnetic beads. mRNA was fragmented by ionic interruption, and 300 bp fragments were used as a template to synthesize the first strand using reverse transcriptase and random hexamer primer. The first strand served as a template for second-strand synthesis. The constructed library was enriched by PCR, and quality of 450 bp fragments was verified by Agilent 2100 Bioanalyzer. After dilution of the individual samples to 2 nM and denaturation to form single-stranded RNA libraries, paired-end sequencing was performed using next-generation sequencing on an Illumina platform (Illumina NovaSeq 6000) (Table S1). Filtered reads (Table S2) were aligned to the reference genome (*A. fumigatus* Af293, NCBI RefSeq assembly GCF_000002655.1) using HISAT2 (Table S3). Alignment files were used to generate read counts by HTseq, and FPKM (fragments per kilobases per million fragments) was used to normalize expression (Fig. S6 and S7). Differential gene expression was analyzed using DESeq. If the *P*-value was below 0.05 and log_2_(fold change) greater than or equal to 1, a gene was considered differentially expressed (Tables S4 to S6; Fig. S8). Bidirectional clustering analysis has been performed using the pheatmap package in R environment. Distances were calculated using the Euclidean method and complete linkage hierarchical clustering using longest distance method (Fig. S9).

GO enrichment analysis was performed using topGO. *P*-value was calculated by the hypergeometric distribution model. A GO term was considered significantly enriched if the *P*-value was below 0.05 (Tables S7 and S8; Fig. S10 and S11). Furthermore, functional KEGG enrichment analysis was performed (Tables S9 and S10; Fig. S12 and S13).

### Virulence assays in *G. mellonella*

*G. mellonella* larvae were purchased from Keyun Biological Co. China. Larvae were placed in a 37°C constant-temperature incubator overnight and retrieved the following day to perform subsequent experiments. Fresh conidia of the indicated *A. fumigatus* strains were harvested from agar plates, suspended in phosphate-buffered saline (PBS; pH 7.2–7.4) and filtered to remove hyphal fragments. Conidia were quantified using a hemocytometer, and the conidial suspensions were adjusted to a concentration of 1 × 10^8^ conidia/mL in sterile PBS. Then, 10 µL conidial suspension was injected into *G. mellonella* larvae (~0.3 g for each) via the left prolegs. As a control group, larvae were injected with PBS, pH 7.2–7.4. After injection, all larvae were incubated at 37°C with humidity levels around 29–33%, and their survival rates were evaluated every 24 h. The procedure was conducted with groups of 20 larvae.

### Histopathology of infected *G. mellonella* larvae

Two representative larvae per group were selected to be fixed in 4% paraformaldehyde and then embedded in optimal cutting temperature compound and sectioned using cryosectioning (frozen sectioning). Sections were then stained with hematoxylin and eosin (H&E) and periodic acid-Schiff-methenamine silver (PASM) separately for histological staining analysis performed by Wuhan Servicebio Technology Co., Ltd.

### Quantification of neosartoricin B

*A. fumigatus* strains were grown in liquid minimal medium at 37 °C in the dark. After 48 hours of static incubation, 10  mL of each liquid culture was harvested and extracted with an equal volume (10  mL) of ethyl acetate/methanol/acetic acid (89:10:1). The organic phase was evaporated to dryness, and the residue was redissolved in 1  mL of methanol. The extracted samples were analyzed using LC-MS/MS on an Agilent 1200 Infinity HPLC system coupled to an Agilent 6410B triple quadrupole mass spectrometer (Agilent Technologies, USA). Chromatographic separation was achieved on a C18 column (100 mm × 2.1 mm, 1.7  µm particle size), with a flow rate of 0.2  mL/min. The mobile phase (30% water and 70% acetonitrile) was delivered under isocratic conditions at a flow rate of 0.2  mL/min, with a 10  µL injection volume. Neosartoricin B was detected in the negative electrospray ionization (ESI^−^) mode using optimized multiple reaction monitoring (MRM) transitions. Two MRM pairs were monitored for quantification and confirmation: *m*/*z* 443.1 → 423.2 and 443.1 → 339.1. Quantification was normalized to the fresh weight of the harvested mycelia.

## Data Availability

The data discussed in this work have been submitted to NCBI. Raw sequencing reads are available at the NCBI database (BioProject ID: PRJNA1198059).

## SUPPLEMENTAL MATERIAL

Supplemental figures mbio.02204-25-s0001.pdf Fig. S1-S5.

Supplemental material mbio.02204-25-s0002.pdf Fig. S6-S13 and supplemental table captions.

Supplemental tables mbio.02204-25-s0003.pdf Tables S1-S4.

Table S5 mbio.02204-25-s0004.xlsx Differentially expressed genes in *A. fumigatus* ∆*fphA* and WT.

Table S6 mbio.02204-25-s0005.xlsx Differentially expressed genes in *A. fumigatus* ∆*fphB* and WT.

Table S7 mbio.02204-25-s0006.xlsx GO enrichment of *A. fumigatus* ∆*fphA* and WT.

Table S8 mbio.02204-25-s0007.xlsx GO enrichment of *A. fumigatus* ∆*fphB* and WT.

Table S9 mbio.02204-25-s0008.xlsx KEGG enrichment of *A. fumigatus* ∆*fphA* and WT.

Table S10 mbio.02204-25-s0009.xlsx KEGG enrichment of *A. fumigatus* ∆*fphB* and WT.

ASM does not own the copyrights to Supplemental Material that may be linked to, or accessed through, an article. The authors have granted ASM a non-exclusive, world-wide license to publish the Supplemental Material files. Please contact the corresponding author directly for reuse.
