# Supplementary material for: Distinct roles of phytochromes A and B in Aspergillus fumigatus in environmental sensing and pathogenicity
Source: mBio. 2025 Sep 23;16(11):e02204-25. doi: 10.1128/mbio.02204-25 (PMC12607886; doi:10.1128/mbio.02204-25)
Supplement: Supplemental figures — Fig. S1-S5. [file mbio.02204-25-s0001.pdf]

Divergent Roles of Phytochromes in *Aspergillus fumigatus:* Phytochrome A Senses Light and Temperature, Phytochrome B Modulates Host Infection in *Galleria mellonella*

Kai Leister[1], Yan Dong[2], Alexander Landmark[1], Yinyan Ma[2], Birgit Schreckenberger[1], Zhenzhong Yu[3], Ling Lu*[2] and Reinhard Fischer[1]*

running head: phytochromes in *A. fumigatus*

Address: [1]Karlsruhe Institute of Technology (KIT) - South Campus
Institute for Applied Biosciences
Dept. of Microbiology
Fritz-Haber-Weg 4
D-76131 Karlsruhe, Germany
Phone: +49-721-6084-4630
Fax: +49-721-6084-4509
E-mail: reinhard.fischer@KIT.edu
Homepage: www.iab.kit.edu

[2]Department of Clinical Laboratory
Nanjing Drum Tower Hospital
College of Life Sciences
Nanjing Normal University
Nanjing, 210023, China
Email: linglu@njnu.edu.cn
Tel:13915997578

[3] Nanjing Agricultural University
Jiangsu Provincial Key Lab for Organic Solid Waste Utilization
Jiangsu Collaborative Innovation Center for Solid Organic Waste Resource Utilization
Educational Ministry Engineering Center of Resource-saving fertilizers
210095 Nanjing, China
E-mail: yuzhenzhong@njau.edu.cn
Homepage: http://cres.njau.edu.cn/info/1155/1652.htm

* Corresponding authors

Key words: phytochrome signaling, *A. nidulans*, *A. fumigatus,* pathogenicity.

Supplemental figures

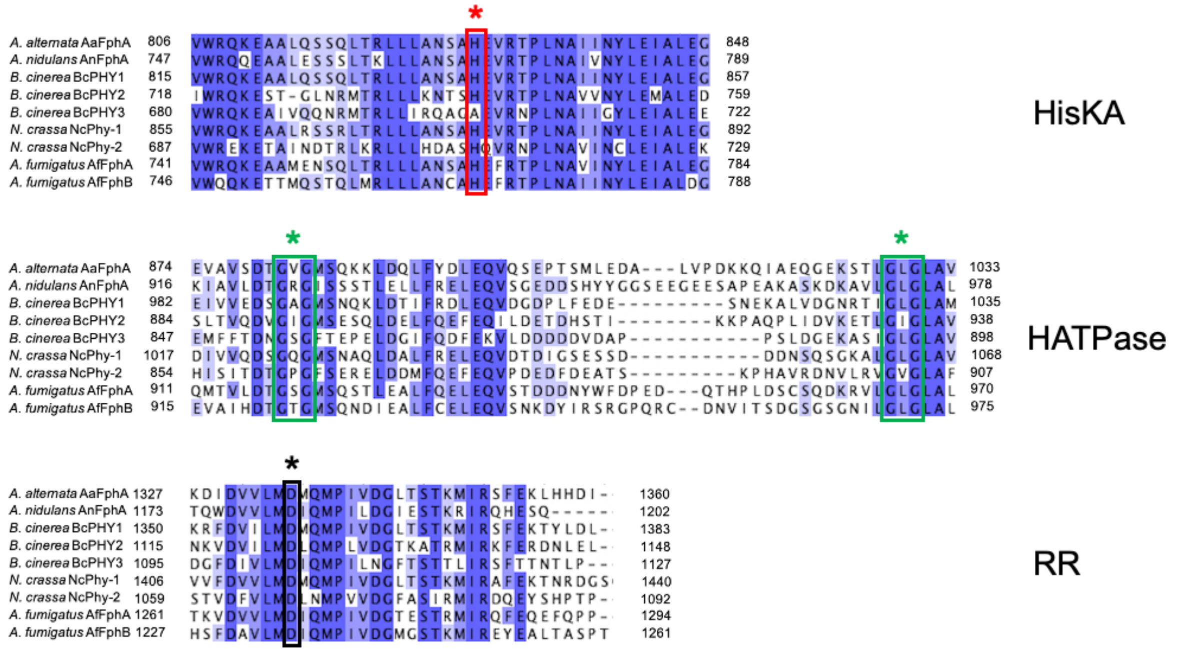

Figure S1: FphA and FphB possess all the characteristics of a functional hybrid-histidine kinase in the regulatory module. Sequence alignments of characterized phytochromes of the phylum ascomycota. Red box and asterisk: conserved histidine of the histidine kinase domain. Green boxes and asterisks: conserved *GxG*-motif flanking the ATP-binding pocket of the ATPase domain. Black box and asterisk: conserved aspartate residue in the response regulator domain.

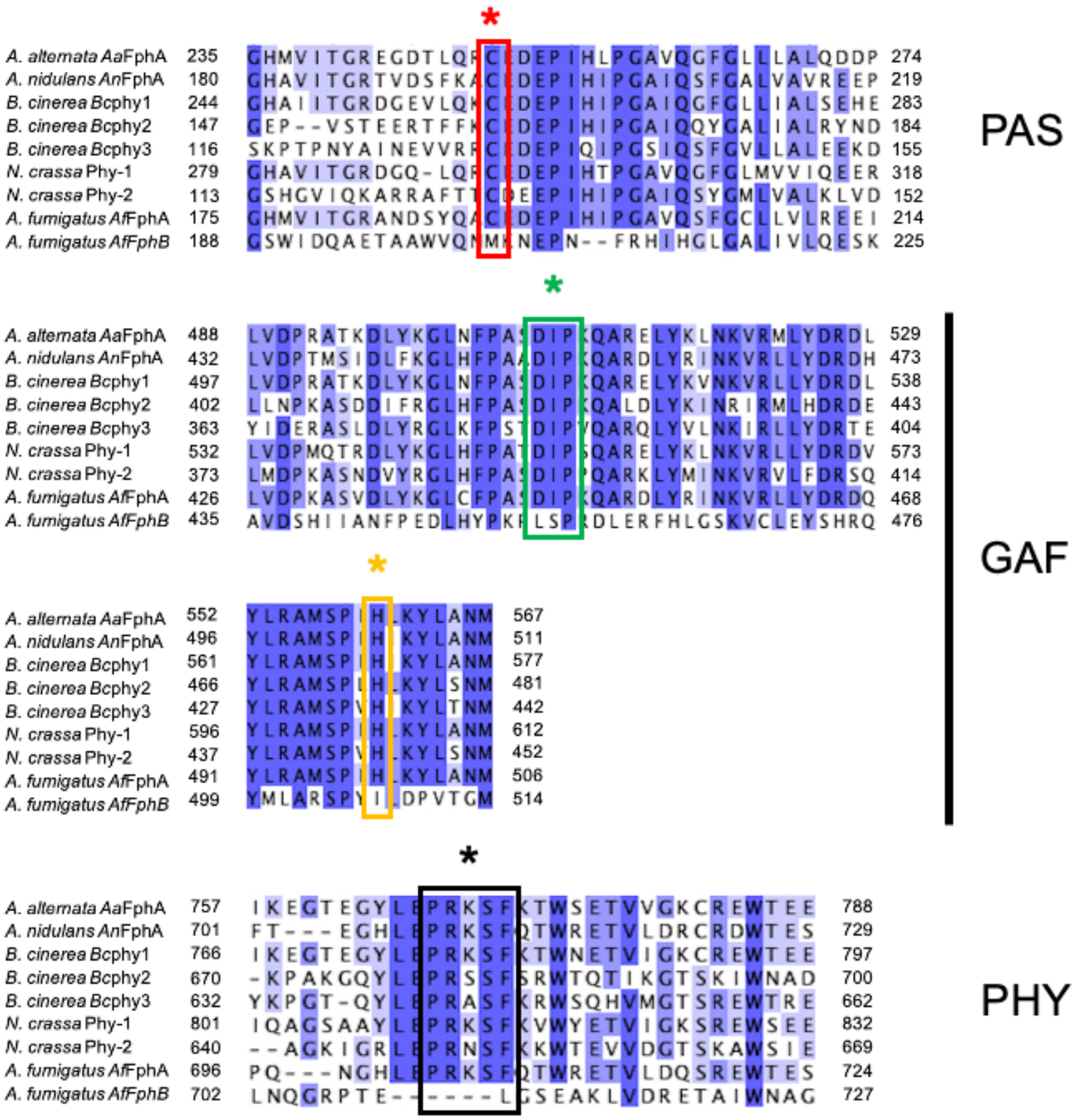

Figure S2: AfFphB lacks critical residues and motifs in the photosensory domain. Sequence alignments of characterized phytochromes of the phylum ascomycota. Red box and asterisk: critical cysteine for chromophore binding. Green box and asterisk: *DIP*-motif, involved in signal transduction to the C-terminal regulatory module. Orange box and asterisk: histidine involved in chromophore coordination. Black box and asterisk: *PRxSF*-motif, involved in signal transduction to the C-terminal regulatory module.

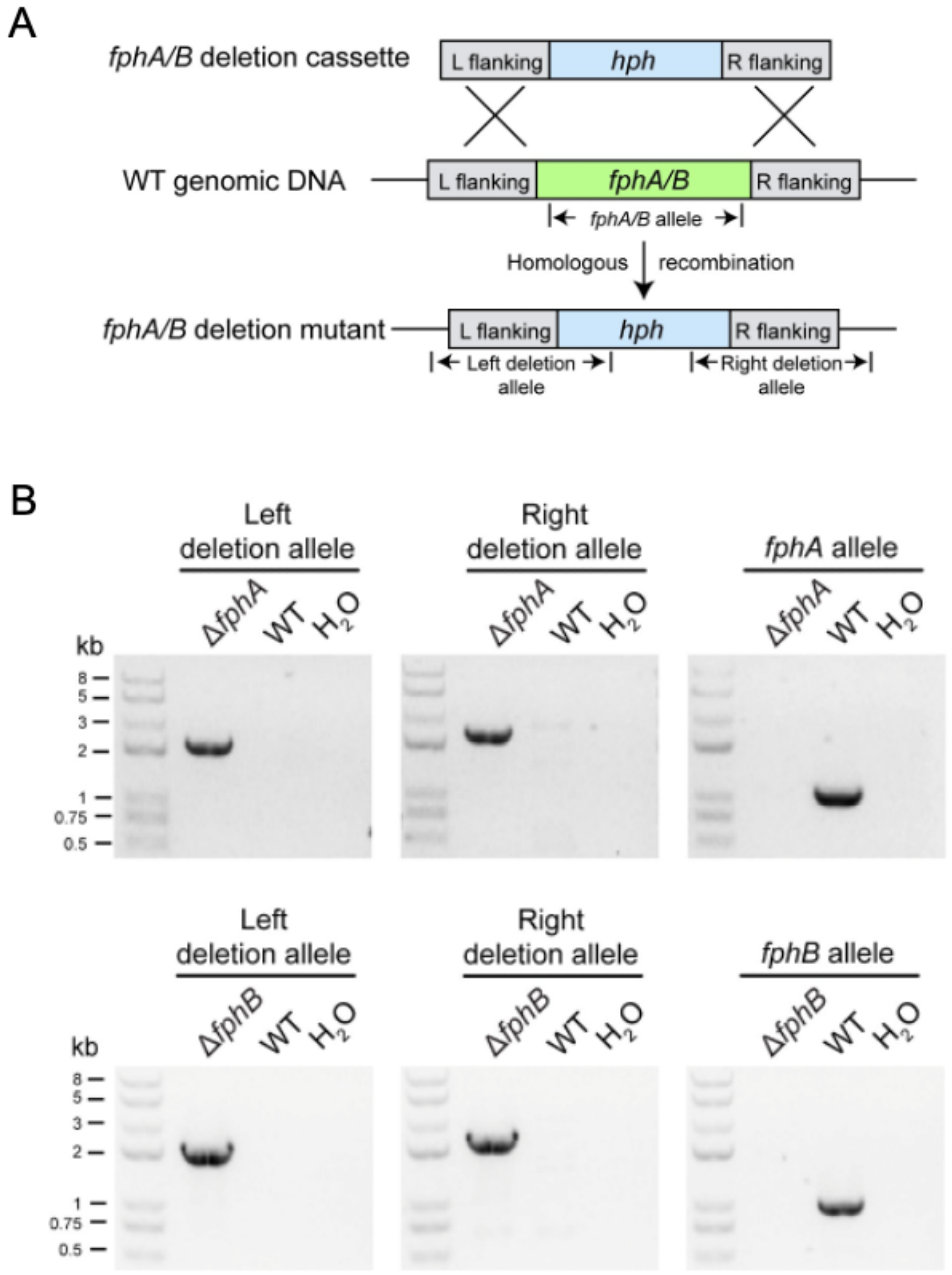

Figure S3: Deletion of *fphA* and *fphB* in *A. fumigatus*. A Gene deletion by homologous recombination. **B** Verification of gene deletions by PCR.

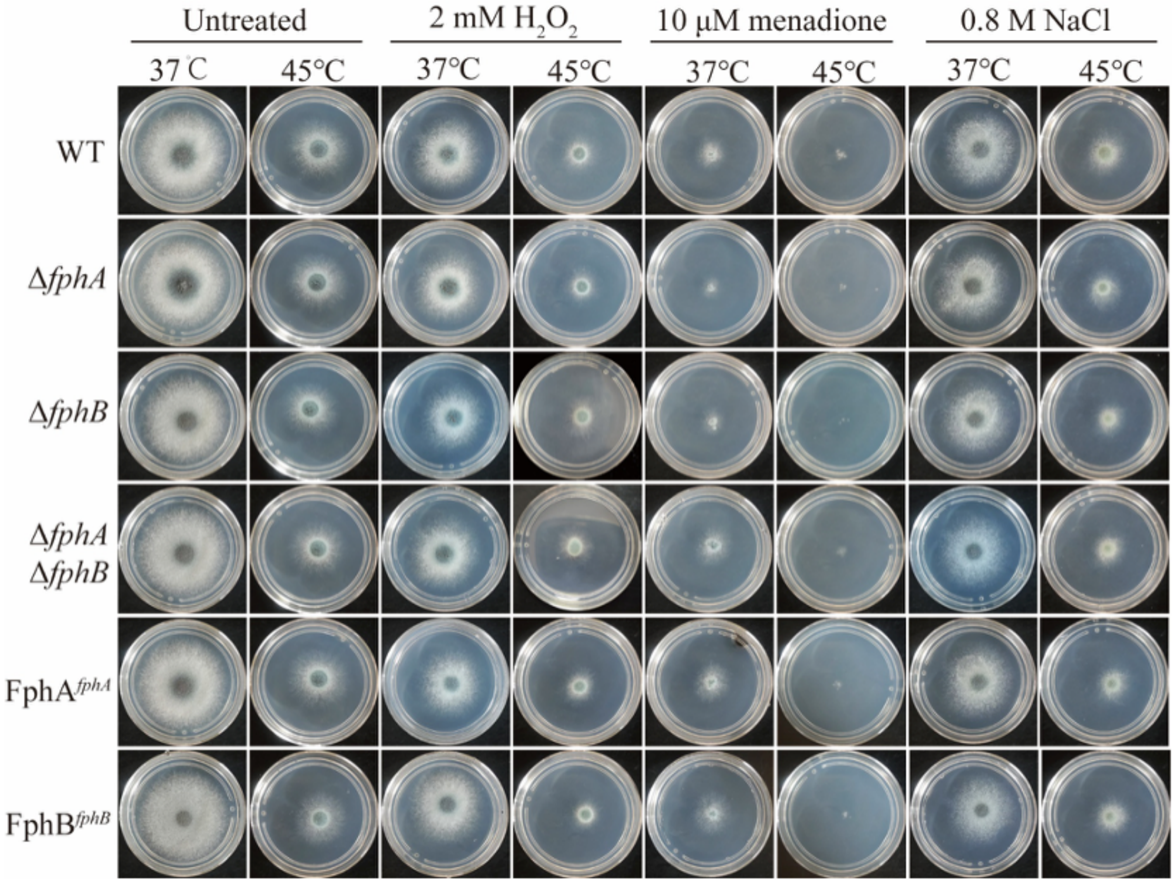

Figure S4: FphA and FphB do not play a role in stress resistance. Colony phenotypes of *A. fumigatus* WT, Δ*fphA,* Δ*fphB* and Δ*fphA*Δ*fphB* and complemented strains at the conditions indicated in the figure.

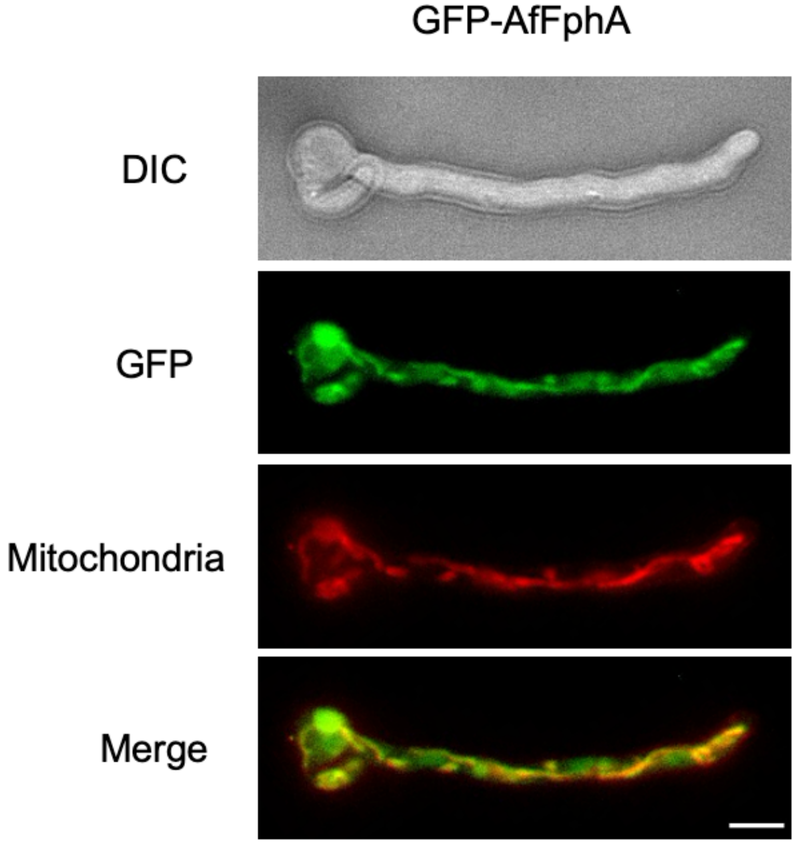

Figure S5: AfFphA localizes at mitochondria. Fluorescence microscopy images of GFP-AfFphA in *A. nidulans*. AfFphA localizes at mitochondria. Mitochondria were stained with MitoTracker™ Red CMXRos (Thermo Fisher Scientific, Waltham, USA).
