# Supplementary material for: Distinct roles of phytochromes A and B in Aspergillus fumigatus in environmental sensing and pathogenicity
Source: mBio. 2025 Sep 23;16(11):e02204-25. doi: 10.1128/mbio.02204-25 (PMC12607886; doi:10.1128/mbio.02204-25)
Supplement: Supplemental material — Fig. S6-S13 and supplemental table captions. [file mbio.02204-25-s0002.pdf]

# Divergent Roles of Phytochromes in *Aspergillus fumigatus:* Phytochrome A Senses Light and Temperature, Phytochrome B Modulates Host Infection in *Galleria mellonella*

Kai Leister[1], Yan Dong[2], Alexander Landmark[1], Yinyan Ma[2], Birgit Schreckenberger[1], Zhenzhong Yu[3], Ling Lu*[2] and Reinhard Fischer[1]*

**running head:** phytochromes in *A. fumigatus*

**Address**:

[1]Karlsruhe Institute of Technology (KIT) - South Campus
Institute for Applied Biosciences
Dept. of Microbiology
Fritz-Haber-Weg 4
D-76131 Karlsruhe, Germany
Phone: +49-721-6084-4630
Fax: +49-721-6084-4509
E-mail: reinhard.fischer@KIT.edu
Homepage: www.iab.kit.edu

[2]Department of Clinical Laboratory
Nanjing Drum Tower Hospital
College of Life Sciences
Nanjing Normal University
Nanjing, 210023, China
Email: linglu@njnu.edu.cn
Tel:13915997578

[3] Nanjing Agricultural University
Jiangsu Provincial Key Lab for Organic Solid Waste Utilization
Jiangsu Collaborative Innovation Center for Solid Organic Waste Resource Utilization
Educational Ministry Engineering Center of Resource-saving fertilizers
210095 Nanjing, China
E-mail: yuzhenzhong@njau.edu.cn
Homepage: http://cres.njau.edu.cn/info/1155/1652.htm

* Corresponding authors

**Key words:** phytochrome signaling, *A. nidulans*, *A. fumigatus,* pathogenicity.

## Supplemental figures

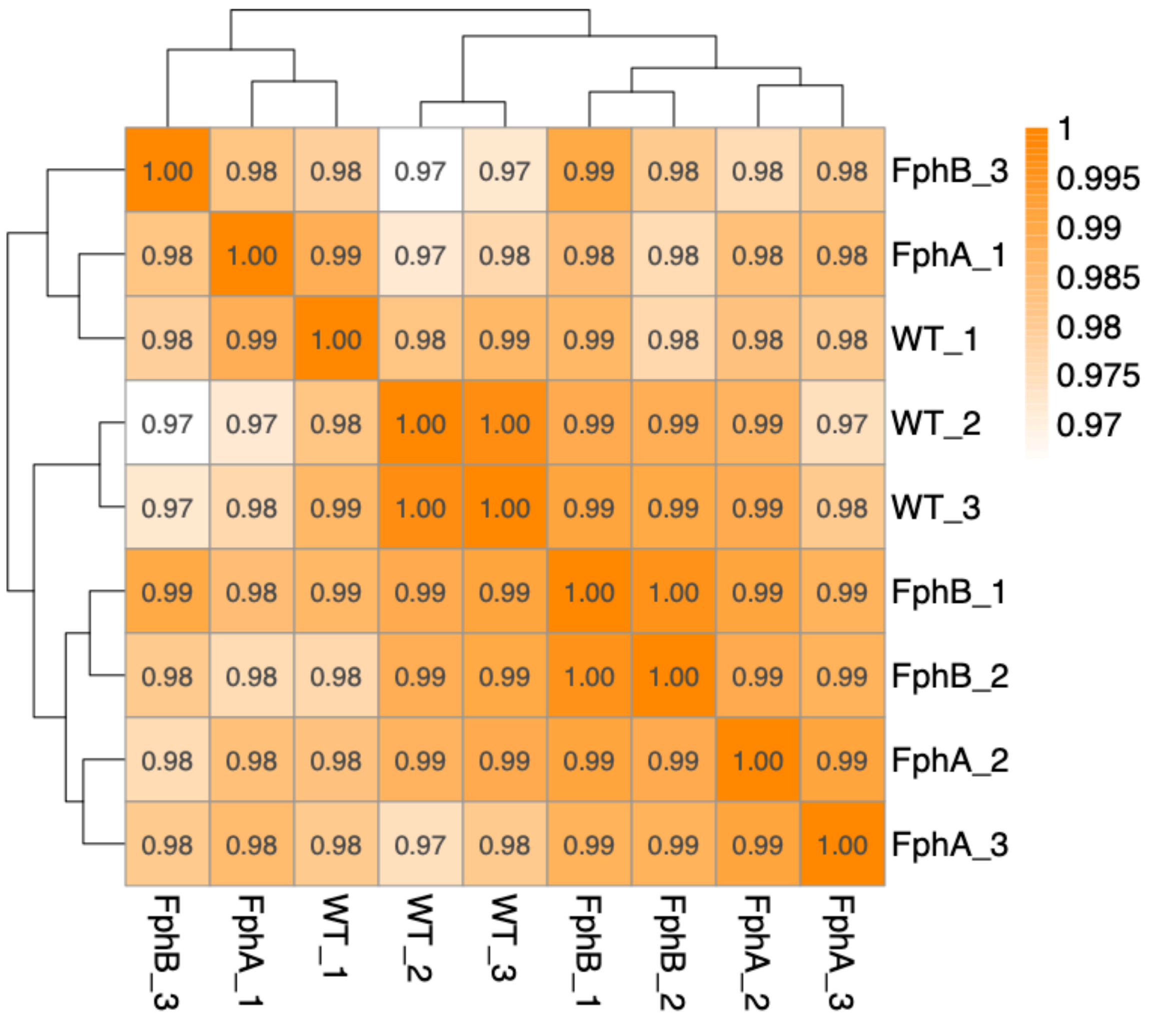


**Figure S6: Gene expression level correlation.** Pearson's correlation coefficient has been used to express the correlation of gene expression levels between all indivual samples.

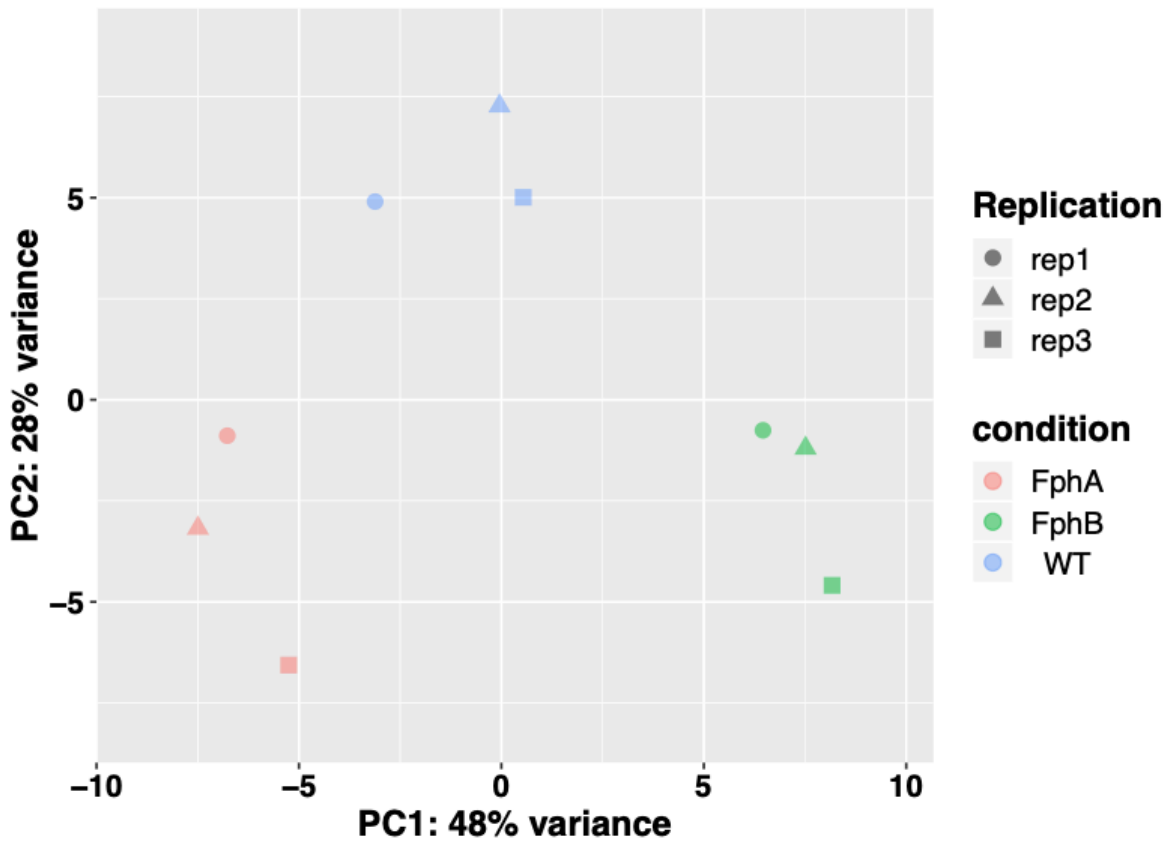


**Figure S7: Principal Components Analysis (PCA).** PCA has been performed using the *DESeq* package in R environment on each individual sample

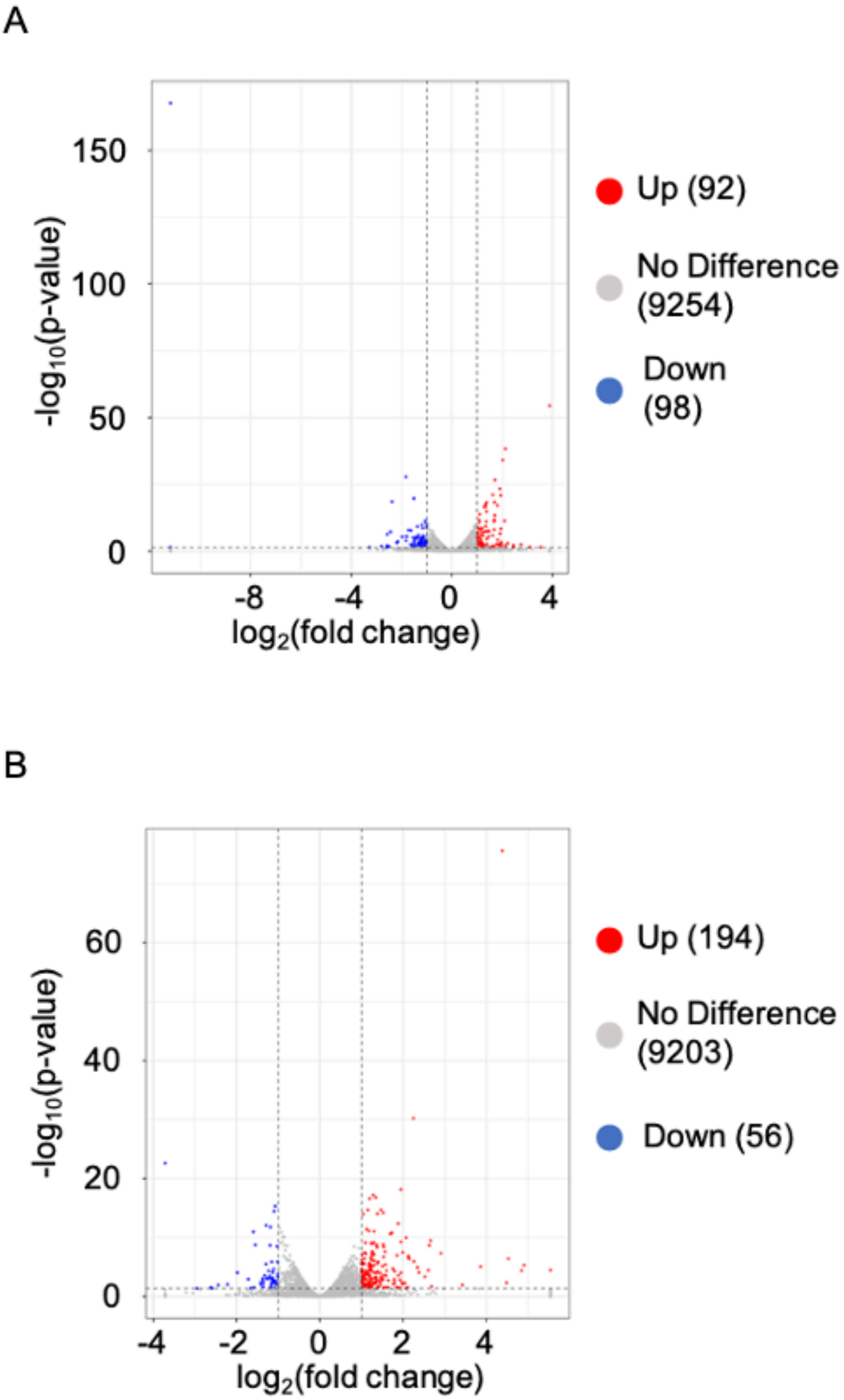


**Figure S8: Distribution of differentially expressed genes.** Volcano plots show the distribution, fold change in expression and significance of differentially expressed genes in *A. fumigatus* Δ*fphA* (A) and Δ*fphB* (B) and WT as control. Volcano plots were generated using *ggplot2* package in R environment.

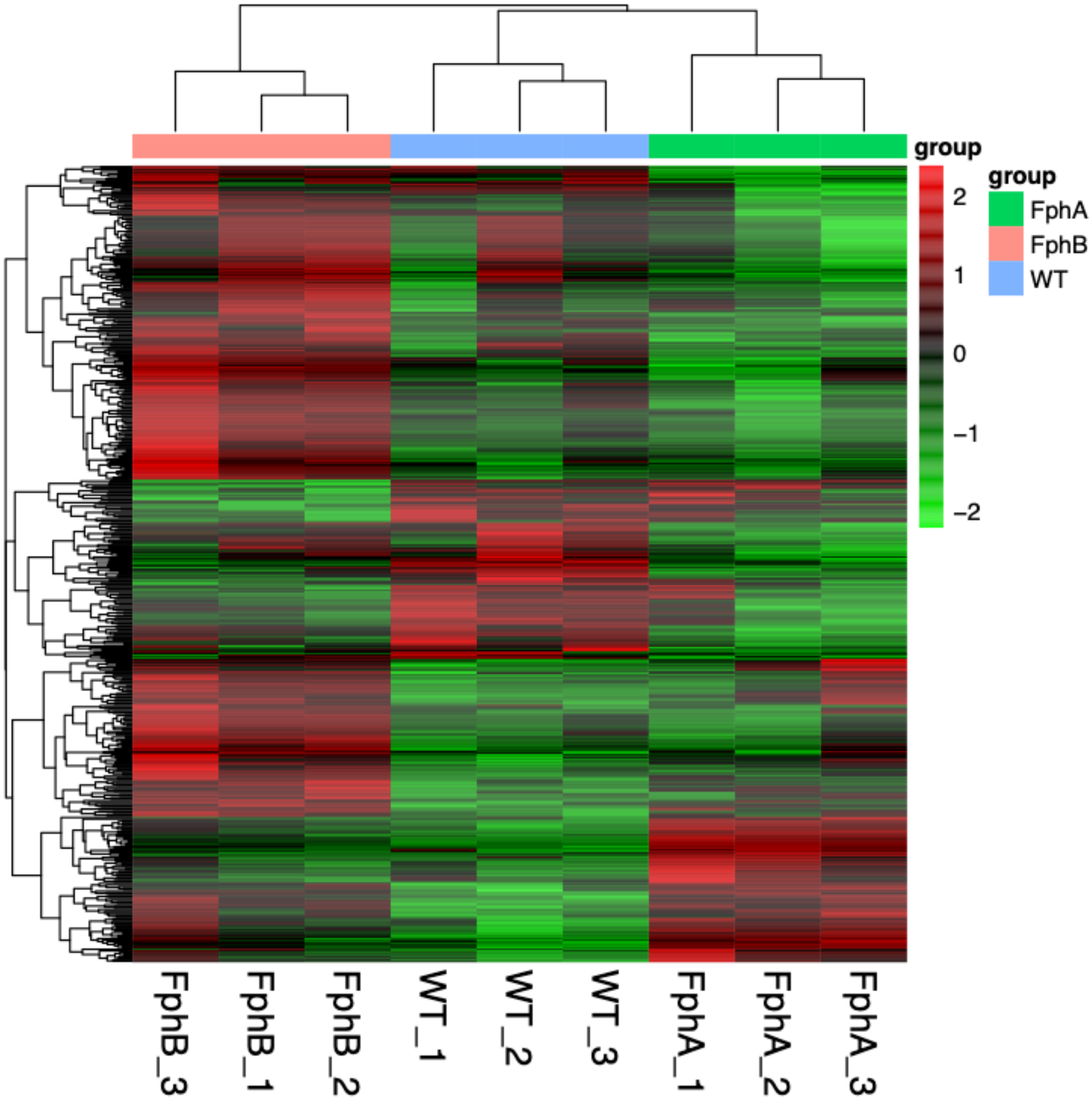


**Figure S9: Cluster analysis of differentially expressed genes.** Bidirectional clustering analysis has been performed using the *pheatmap* package in R environment. Distances were calculated using the Euclidian method and complete linkage hierarchical clustering using longest distance method.

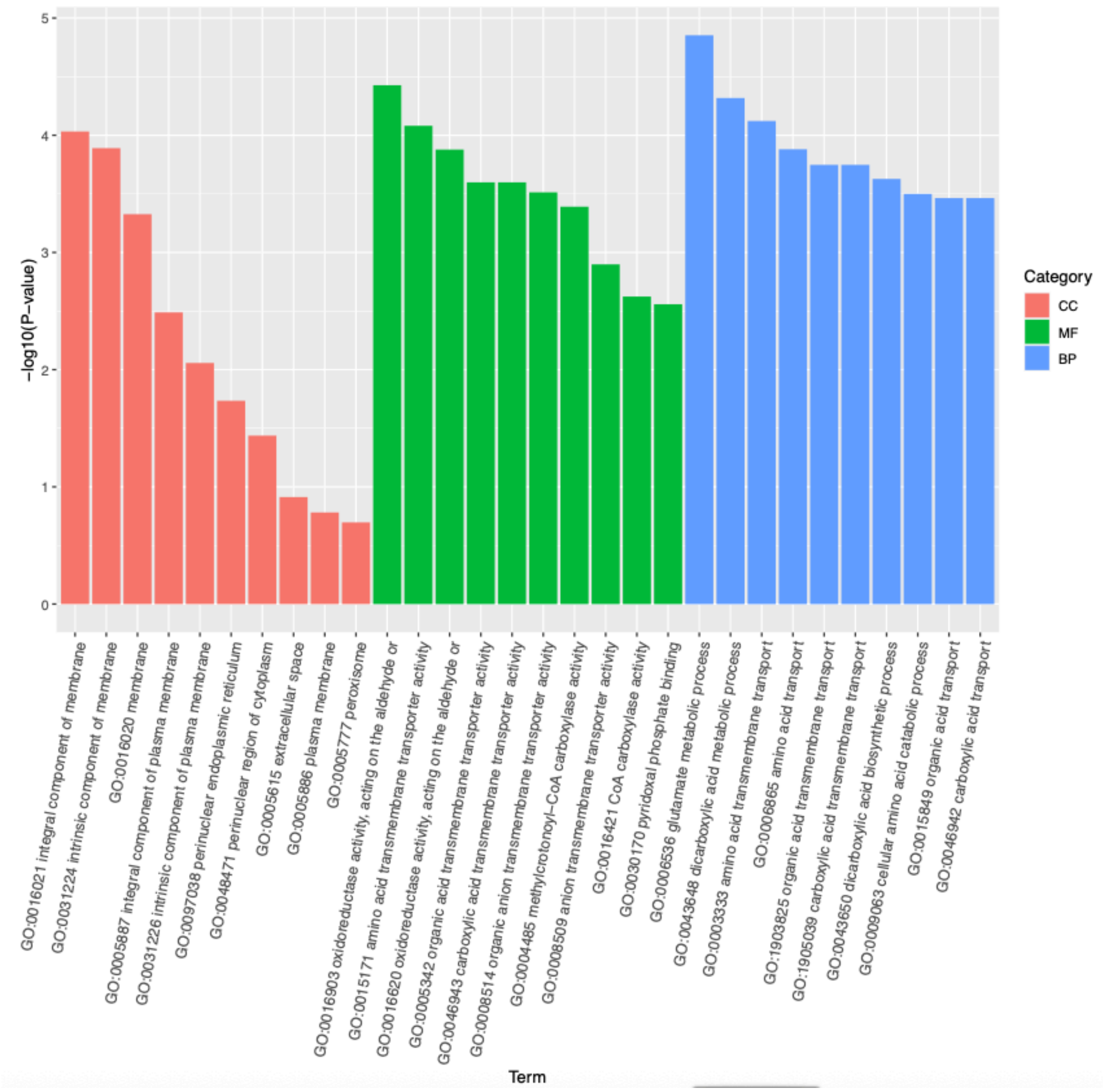


**Figure S10: GO enrichment in *A. fumigatus* ∆*fphA*.** Barplot of enriched GO terms in *A. fumigatus* ∆*fphA*. Top 10 GO terms of the categories "cellular component" (CC), "molecular function" (MF) and "biological process" (BP) displaying the lowest p-values were selected. *A. fumigatus* WT served as control.

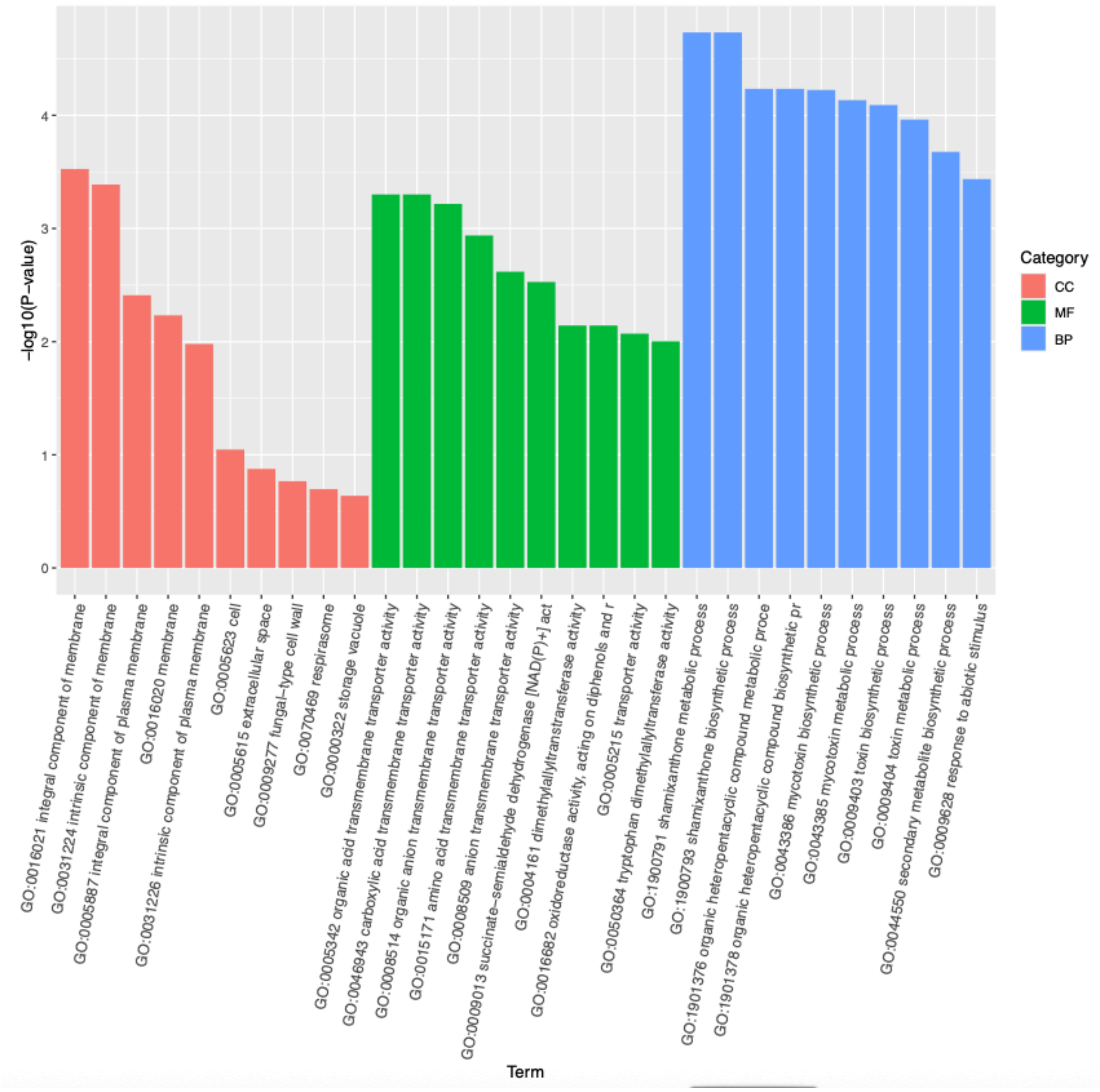


**Figure S11: GO enrichment in *A. fumigatus* ∆*fphB*.** Barplot of enriched GO terms in *A. fumigatus* ∆*fphA*. Top 10 GO terms of the categories “cellular component” (CC), “molecular function” (MF) and “biological process” (BP) displaying the lowest p-values were selected. *A. fumigatus* WT served as control.

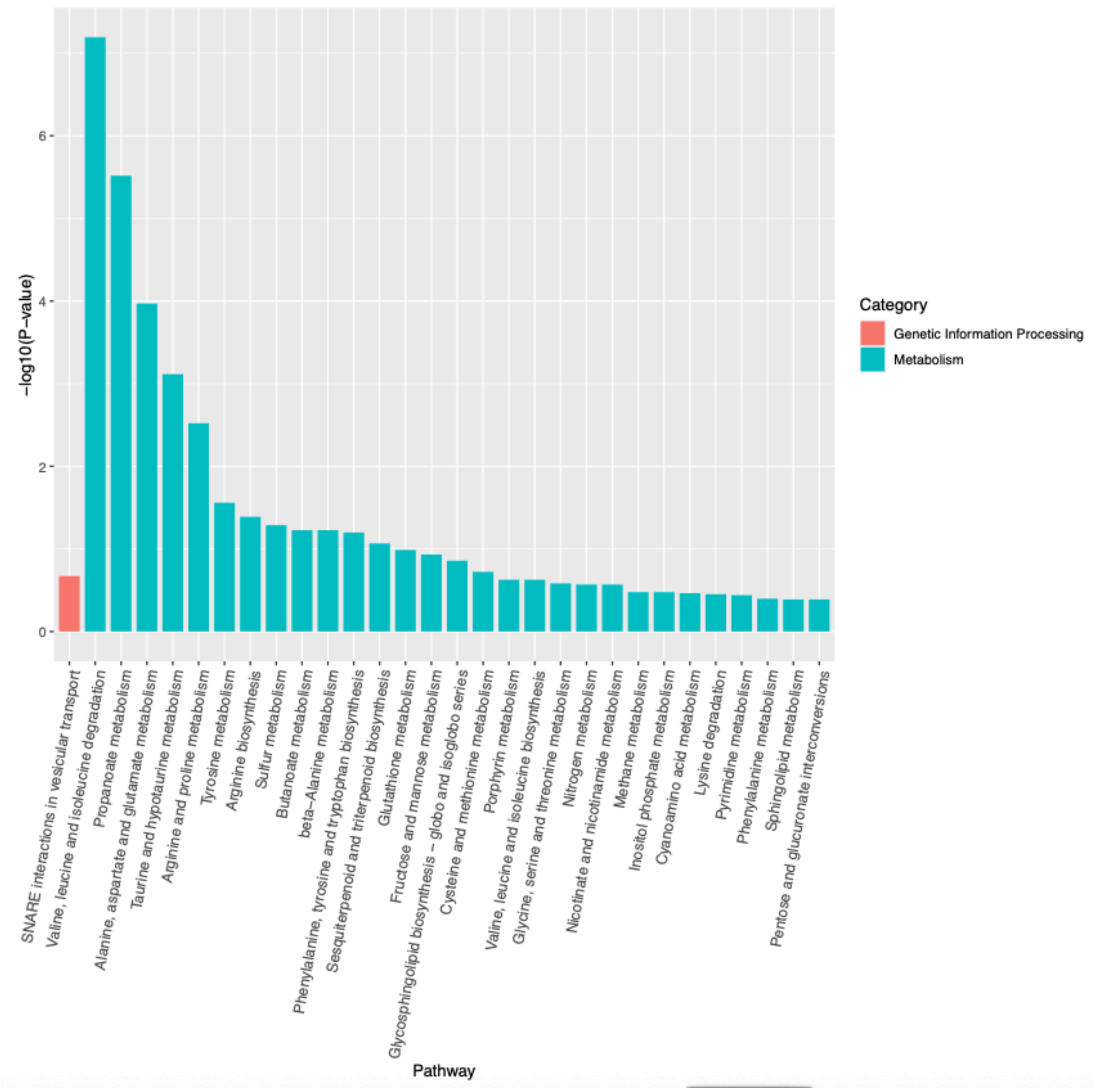


**Figure S12: KEGG pathway enrichment in *A. fumigatus* Δ*fphA*.** Barplot of enriched KEGG pathways in *A. fumigatus* Δ*fphA*. Top 30 KEGG pathways displaying the lowest p-values were selected. *A. fumigatus* WT served as control.

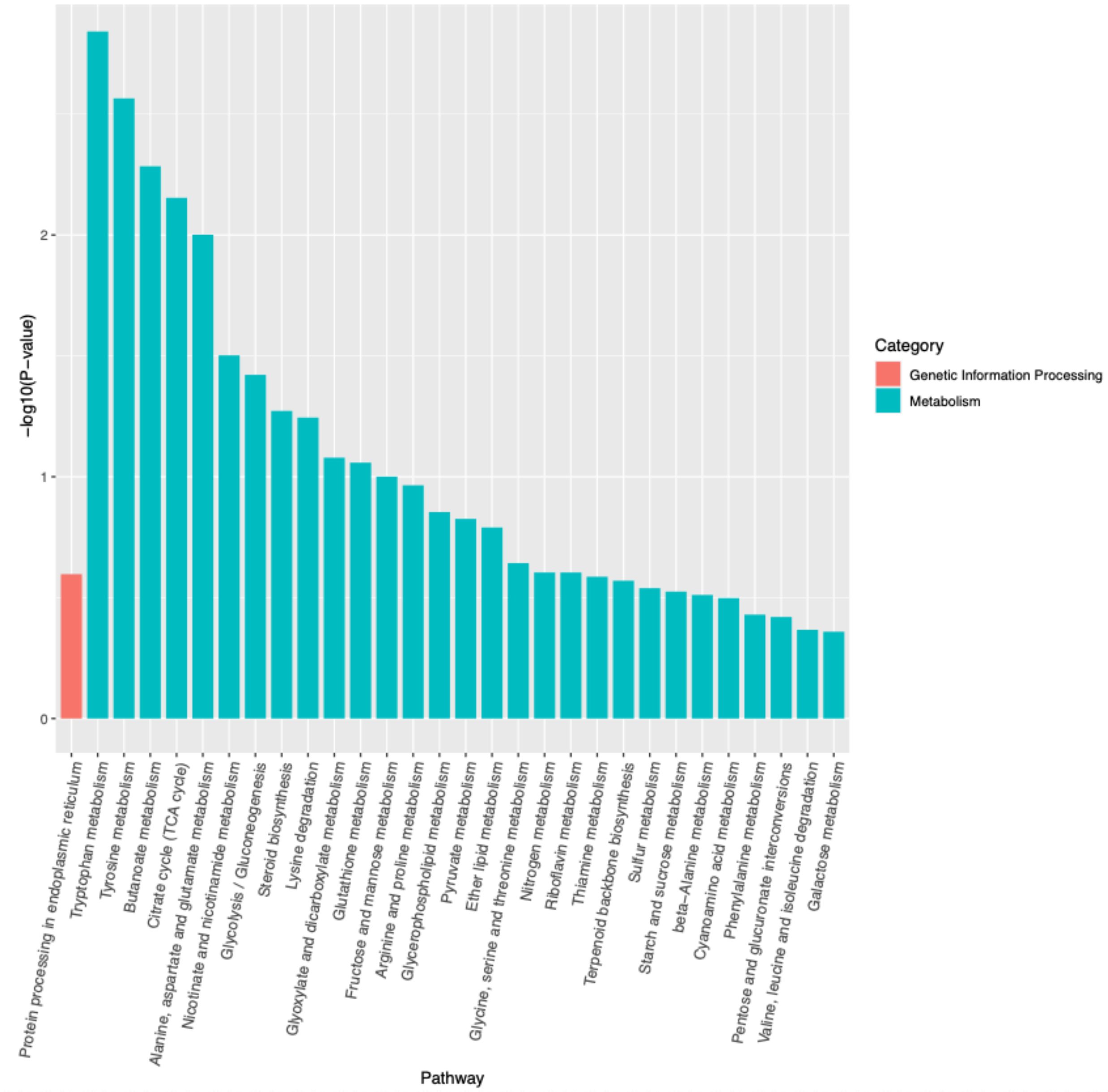


**Figure S13: KEGG pathway enrichment in *A. fumigatus* ∆*fphB*.** Barplot of enriched KEGG pathways in *A. fumigatus* ∆*fphA*. Top 30 KEGG pathways displaying the lowest p-values were selected. *A. fumigatus* WT served as control.

**Supplemental Tables**

**Table S1: Statistic of raw data.** Statistics on the raw data of each individual sample has been performed, including Q30, percentage of ambiguous bases, Q20 (%) and Q30 (%).

**Table S2: Statistics of data filtering.** Sequencing data has been filtered to remove low-quality reads. Criteria of data filtering: 1) Removal of sequences with adaptors at the 3' end. 2) Removal of reads with average quality scores lower than Q20.

**Table S3: Statistics of mapping results.** Filtered reads were mapped to the reference genome using HISAT2

**Table S4: Statistics of differential gene expression analysis.** *DESeq* has been used for analysis of differential gene expression analysis. A gene was considered as differentially expressed if $\log_2$(fold change) ≥ 1 and $p < 0.05$.

**Table S5: List of differentially expressed genes in *A. fumigatus* ∆*fphA* and *A. fumigatus* WT as control.** *DESeq* has been used for analysis of differential gene expression analysis. A gene was considered as differentially expressed if $\log_2$(fold change) ≥ 1 and $p < 0.05$.

**Table S6: List of differentially expressed genes in *A. fumigatus* ∆*fphB* and *A. fumigatus* WT as control.** *DESeq* has been used for analysis of differential gene expression analysis. A gene was considered as differentially expressed if $\log_2$(fold change) ≥ 1 and $p < 0.05$.

**Table S7: GO enrichment of *A. fumigatus* ∆*fphA* and *A. fumigatus* WT as control.** List of enriched GO terms and corresponding genes.

**Table S8: GO enrichment of *A. fumigatus* ∆*fphB* and *A. fumigatus* WT as control.** List of enriched GO terms and corresponding genes.

**Table S9: KEGG enrichment of *A. fumigatus* ∆*fphA* and *A. fumigatus* WT as control.** List of enriched GEGG pathways and corresponding genes.

**Table S10: KEGG enrichment of *A. fumigatus* ∆*fphB* and *A. fumigatus* WT as control.** List of enriched GEGG pathways and corresponding genes.
