# Supplementary material for: Distinct roles of phytochromes A and B in Aspergillus fumigatus in environmental sensing and pathogenicity
Source: mBio. 2025 Sep 23;16(11):e02204-25. doi: 10.1128/mbio.02204-25 (PMC12607886; doi:10.1128/mbio.02204-25)
Supplement: Supplemental tables — Tables S1-S4. [file mbio.02204-25-s0003.pdf]

Table S1: Statistics of raw data

Sample	Reads No.	Bases (bp)	Q30 (bp)	N (%)	Q20 (%)	Q30 (%)
FphA_1	46917020	7084470020	6795534369	0.002416	97.76	95.92
FphA_2	41627010	6285678510	6021954637	0.002398	97.74	95.80
FphA_3	44422640	6707818640	6448888509	0.002403	97.88	96.14
FphB_1	41770196	6307299596	6065148646	0.002408	97.90	96.16
FphB_2	49216506	7431692406	7156595666	0.002413	97.97	96.30
FphB_3	45393832	6854468632	6576672783	0.002423	97.79	95.95
WT_1	47913568	7234948768	6950941052	0.002420	97.86	96.07
WT_2	42562940	6427003940	6169862600	0.002420	97.81	96.00
WT_3	42722982	6451170282	6204160910	0.002380	97.91	96.17

Sample	Sample name
Reads No.	Total number of reads
Bases (bp)	Total number of bases
Q30 (bp)	Total number of bases with 99.9% or more base identification accuracy
N (%)	Percentage of ambiguous bases
Q20 (%)	Percentage of bases with 99% or greater base identification accuracy
Q30 (%)	Percentage of bases with 99.9% or greater base identification accuracy

Table S2: Data filtering statistics

Criteria: 1) Removing of sequences with an adaptor at the 3' end using Fastp

2) Removal of reads with an average quality score below Q20

**Sample**	**Clean Reads**	**Clean Data (bp**	**Clean Rea**	**Clean Data (%)**
FphA_1	45915982	6910036261	97.87	97.54
FphA_2	40547232	6101604040	97.41	97.07
FphA_3	43606332	6563127279	98.16	97.84
FphB_1	40981868	6167247630	98.11	97.78
FphB_2	48353952	7274719681	98.25	97.89
FphB_3	44457966	6689214816	97.94	97.59
WT_1	46972504	7080955532	98.04	97.87
WT_2	41684398	6273604548	97.94	97.61
WT_3	41909308	6307758210	98.10	97.78

Sample	Sample name
Clean Read	Number of high-quality sequence reads
Clean Data	Number of high-quality sequence bases
Clean Read	Percentage of high-quality sequence reads
Clean Data	Percentage of high-quality sequence bases

Table S3: Statistics of mapping results

Sample	Clean_Rea	Total_Mapp	Multiple_M	Uniquely_Mapped
FphA_1	45915982	44462837 (	554772 (1.2	43908065 (98.75%)
FphA_2	40547232	39345448 (	496058 (1.2	38849390 (98.74%)
FphA_3	43606332	42185662 (	512987 (1.2	41672675 (98.78%)
FphB_1	40981868	39786656 (	483244 (1.2	39303412 (98.79%)
FphB_2	48353952	46961457 (	569539 (1.2	46391918 (98.79%)
FphB_3	44457966	43130634 (	558045 (1.2	42572589 (98.71%)
WT_1	46972504	45793771 (	676116 (1.4	45117655 (98.52%)
WT_2	41684398	40669170 (	553965 (1.3	40115205 (98.64%)
WT_3	41909308	40886333 (	569210 (1.3	40317123 (98.61%)

Sample	Sample name
Clean Read	Total number of sequences used for mapping
Total Mapp	Total number of sequences in the reference genome on the mapping (percentage: Total_Mapped/Clean_Reads)
Multiple Ma	Total number of sequences mapped to multiple positions (percentage: Multiple_Mapped/Total_Mapped)
Uniquely M	Total number of sequences mapped to only one position (percentage Uniquely_Mapped/Total_Mapped)

Table S4: DEG statistics

Control	Treat	Up-regulated	Down-regulate(	Total
FphA	FphB	245	46	291
WT	FphB	194	56	250
WT	FphA	92	98	190
